# Genome Wide Single Locus Single Trait, Multi-Locus and Multi-Trait Association Mapping for Some Important Agronomic Traits in Common Wheat (*T*. *aestivum* L.)

**DOI:** 10.1371/journal.pone.0159343

**Published:** 2016-07-21

**Authors:** Vandana Jaiswal, Vijay Gahlaut, Prabina Kumar Meher, Reyazul Rouf Mir, Jai Prakash Jaiswal, Atmakuri Ramakrishna Rao, Harindra Singh Balyan, Pushpendra Kumar Gupta

**Affiliations:** 1 Department of Genetics and Plant Breeding, Ch. Charan Singh University, Meerut, India; 2 Centre for Agricultural Bioinformatics, Indian Agricultural Statistics Research Institute, New Delhi, India; 3 Dept of Genetics & Plant Breeding, G.B. Pant University of Agriculture & Technology, Pantnagar, India; National Institute of Plant Genome Research, INDIA

## Abstract

Genome wide association study (GWAS) was conducted for 14 agronomic traits in wheat following widely used single locus single trait (SLST) approach, and two recent approaches viz. multi locus mixed model (MLMM), and multi-trait mixed model (MTMM). Association panel consisted of 230 diverse Indian bread wheat cultivars (released during 1910–2006 for commercial cultivation in different agro-climatic regions in India). Three years phenotypic data for 14 traits and genotyping data for 250 SSR markers (distributed across all the 21 wheat chromosomes) was utilized for GWAS. Using SLST, as many as 213 MTAs (p ≤ 0.05, 129 SSRs) were identified for 14 traits, however, only 10 MTAs (~9%; 10 out of 123 MTAs) qualified FDR criteria; these MTAs did not show any linkage drag. Interestingly, these genomic regions were coincident with the genomic regions that were already known to harbor QTLs for same or related agronomic traits. Using MLMM and MTMM, many more QTLs and markers were identified; 22 MTAs (19 QTLs, 21 markers) using MLMM, and 58 MTAs (29 QTLs, 40 markers) using MTMM were identified. In addition, 63 epistatic QTLs were also identified for 13 of the 14 traits, flag leaf length (FLL) being the only exception. Clearly, the power of association mapping improved due to MLMM and MTMM analyses. The epistatic interactions detected during the present study also provided better insight into genetic architecture of the 14 traits that were examined during the present study. Following eight wheat genotypes carried desirable alleles of QTLs for one or more traits, WH542, NI345, NI170, Sharbati Sonora, A90, HW1085, HYB11, and DWR39 (Pragati). These genotypes and the markers associated with important QTLs for major traits can be used in wheat improvement programs either using marker-assisted recurrent selection (MARS) or pseudo-backcrossing method.

## Introduction

Genetic analysis of quantitative traits (QTs) mainly involves either the linkage-based interval mapping or the linkage disequilibrium (LD)-based genome-wide association studies (GWAS). GWAS utilizes diverse germplasm (representing most of the genetic variability), which is the product of hundreds of recombination cycles, thus providing higher resolution of QTL regions [1]. This approach is based on the principle of LD, which if maintained over many generations suggests tight linkage. Sometimes LD may also arise due to reasons other than linkage, which may lead to a large proportion of false-positives. However, statistical options are now available for dealing with such cases [2]. GWAS for yield and related traits have been conducted in several crops [3–6] leading to successful identification of a fairly large number of QTLs for yield-related traits. In a detailed study, in the model plant species, *Arabidopsis thaliana* also, in one of the several GWA studies, MTAs for 107 phenotypes were detected [7], thus demonstrating the utility of GWAS. GWA mapping in wheat has been successfully utilized for identification of QTLs for a number of agronomic traits including the following: 1,000-kernel weight, protein content, sedimentation value, test weight, and starch concentration, plant height, days to heading [8–12], kernel size and milling quality [13], HMW glutenin content [14], disease resistance [15–17], earliness [18], drought adaptive traits and yield [19–21], and pre-harvest sprouting tolerance (PHST) [22–24], etc. GWA mapping has also been utilized for discovery of marker-trait associations and candidate genes for morphological traits in *Ae*. *tauschii*, the donor of the wheat subgenome D [25].

Earlier, in our laboratory, we used SSRs for QTL analysis in wheat using both, interval mapping and single locus single trait (SLST) association mapping [11, 22, 26–27]. SLST is the simplest and most widely used association mapping approach. However, it has been argued that SLST approach for GWAS leads to biased results possibly due to the following reasons: (i) confounding effect of background QTL/genes, (ii) pleiotropism involving control of more than one trait by the same gene/QTL, and (iii) LD for reasons other than linkage. Therefore, multi-locus mixed model (MLMM) and multi-trait mixed model (MTMM) have been proposed to address the issues of background noise and pleiotropy [28–29]. MLMM takes into account genetic background in the same manner as composite interval mapping (CIM) does in case of interval mapping [28]. Similarly, MTMM is comparable to multi-trait QTL interval mapping, and allows detection of individual QTLs that are pleiotropic, although in some cases this may be due to tight linkage also [29]. Epistasis is another issue that has generally been neglected in GWAS. The present communication reports the results of GWAS for 14 traits in common wheat following not only SLST, but also MLMM, MTMM; epistatic interactions are also included. An effort was also made to compare the efficiency of the above three approaches for identification of reliable MTAs in wheat for marker-assisted selection (MAS).

## Materials and Methods

### Association mapping panel and SSR markers

The association mapping panel comprised 230 Indian wheat cultivars (for details, see Mir et al. [6, 29]), released for commercial cultivation in different agro-climatic regions of India during a period of ~100 years (1910 to 2006). These cultivars represented a fairly diverse set of genotypes, as demonstrated in our earlier diversity analysis study [30]. The seed of cultivars was procured from the ICAR-Indian Institute of Wheat and Barley Research (ICAR-IIWBR), Karnal (India). A set of 250 SSR markers spread over all the 21 wheat chromosomes was used for genotyping of the association mapping panel (for details, see Jaiswal et al. [22]).

### Data on 14 agronomic traits

The data on mean values for each of the 14 traits of the above 230 Indian common wheat cultivars (based on trials conducted over three years) was procured from ICAR-IIWBR, Karnal, India [31]; the data procured was subjected to further statistical analysis during the present study. The 14 traits included the following: plant height (PH), peduncle length (PL), flag leaf length (FLL), awn length (AL), days to heading (DTH), days to maturity (DTM), spike length (SL), number of spikelets/spike (SKS), number of grains/spike (GS) and 1000-grain weight (TGW), grain protein content (GPC), hardness index (HI), hectoliter weight (HW) and sedimentation volume (SV).

### Statistical analysis

#### Descriptive statistics for phenotypic trait and structure analysis

Descriptive statistics including frequency distribution, mean values, coefficient of variability (CV) and Pearsons’s correlation coefficients were obtained using SPSS version 17.0. Model-based cluster analysis of association mapping panel was conducted during an earlier study in our lab [11] to infer population structure using the software STRUCTURE version 2.2 [32].

#### Population structure and model selection for MTAs

Multiple regression analysis was carried out to estimate r^2^ (%) and the probability values for determining relationships between the phenotypic traits and population structure [19]. Based on this information, out of the four models including naive, Q, K and Q+K (for details of the models, see section on MTA analysis), the best fit model was selected for each trait following Stich et al. [33]. Following two criteria were used for model selection: (i) lowest mean of squared differences (MSD) between observed and expected p values involving all marker loci, and (ii) percentage of observations being below nominal level (α = 0.05) in a p (expected)—p (observed) plot (quantile-quantile or Q-Q plot). Consequently, different models were used for different traits.

#### Marker-traits association (MTA) analysis

For MTA analysis, marker alleles with frequency ≤ 0.05 were treated as rare and the rare variant genotypes carrying these rare alleles were excluded from the analysis for statistical reasons; the genotypes excluded from the analysis differed for different SSRs. TASSEL version 3.0 (http://www.maizegenetics.net) was used to conduct SLST association mapping—involving associations of individual markers with each of the 14 traits, employing one of the following four models for individual traits: (i) general linear model (GLM: naive model), (ii) GLM including Q-matrix derived from STRUCTURE (Q-model), (iii) the mixed linear model (MLM) based on the kinship matrix (K-model) and (iv) the MLM based on both the Q-matrix and the kinship matrix (Q+K-model) (for more details, see Results). The kinship-matrix was generated by TASSEL through conversion of the distance matrix derived from TASSEL’s cladogram function into a similarity matrix; also the option EMMA was chosen for MLM [34], leaving the other parameters at the default settings. Significance of MTAs was determined at p ≤ 0.05.

In addition to SLST analysis, GWAS using MLMM [28] and MTMM [29] was also conducted. For MLMM, background genome was considered as a cofactor (like CIM in interval mapping) using stepwise mixed-model regression with forward inclusion and backward elimination [28]. For MTMM, all pairs of traits showing significant and strong correlation (p-value≤ 0.05; r^2^ ≥0.25) were used. In MTMM, following three tests were applied: (i) full test that compared the full model including the effect of a marker genotype and its interactions, with the model that included neither, (ii) interaction effect test that compares the full model to one, which does not include interactions, and (iii) common effect test that compares a model with a marker genotype to the model that does not include marker genotype [29].

In each of the above approaches, corrections were made using false discovery rate (FDR) criteria earlier suggested [35] to reduce the proportion of false positives originating due to multiple testing. Since average LD in wheat is 10 cM [36], more than one MTAs within a range of 10 cM were considered to represent the same QTL.

For each trait, two dimensional epistatic interactions were also examined using MTAs detected through SLST, MLMM and MTMM. This analysis was carried out using the function *interactionPva*l available in *SNPassoc* package of R-software [37]. In order to control confounding due to population structure, different corrections (like Q, K or Q+K) were applied for different traits (see later) into the interaction model.

### Identification of desirable QTL alleles and donor genotypes for wheat improvement

QTLs that were detected by all the three methods or by at least two methods were considered to be relatively more important. However, QTLs that were detected by SLST alone and qualified FDR or those reported in earlier literature were also considered important. For identification of desirable QTL alleles, for each trait, a set of 20 genotypes with their superior phenotypic performance was selected. Marker allele for individual marker loci and pairs of alleles for the interacting epistatic loci present in maximum number of genotypes (out of 20 superior genotypes) were taken to be associated with desirable QTL allele for the trait concerned. The corresponding genotypes carrying desirable QTL alleles and a desirable trait value were treated as superior genotypes for individual traits.

## Results

### Descriptive statistics for 14 traits

**Fig 1:**
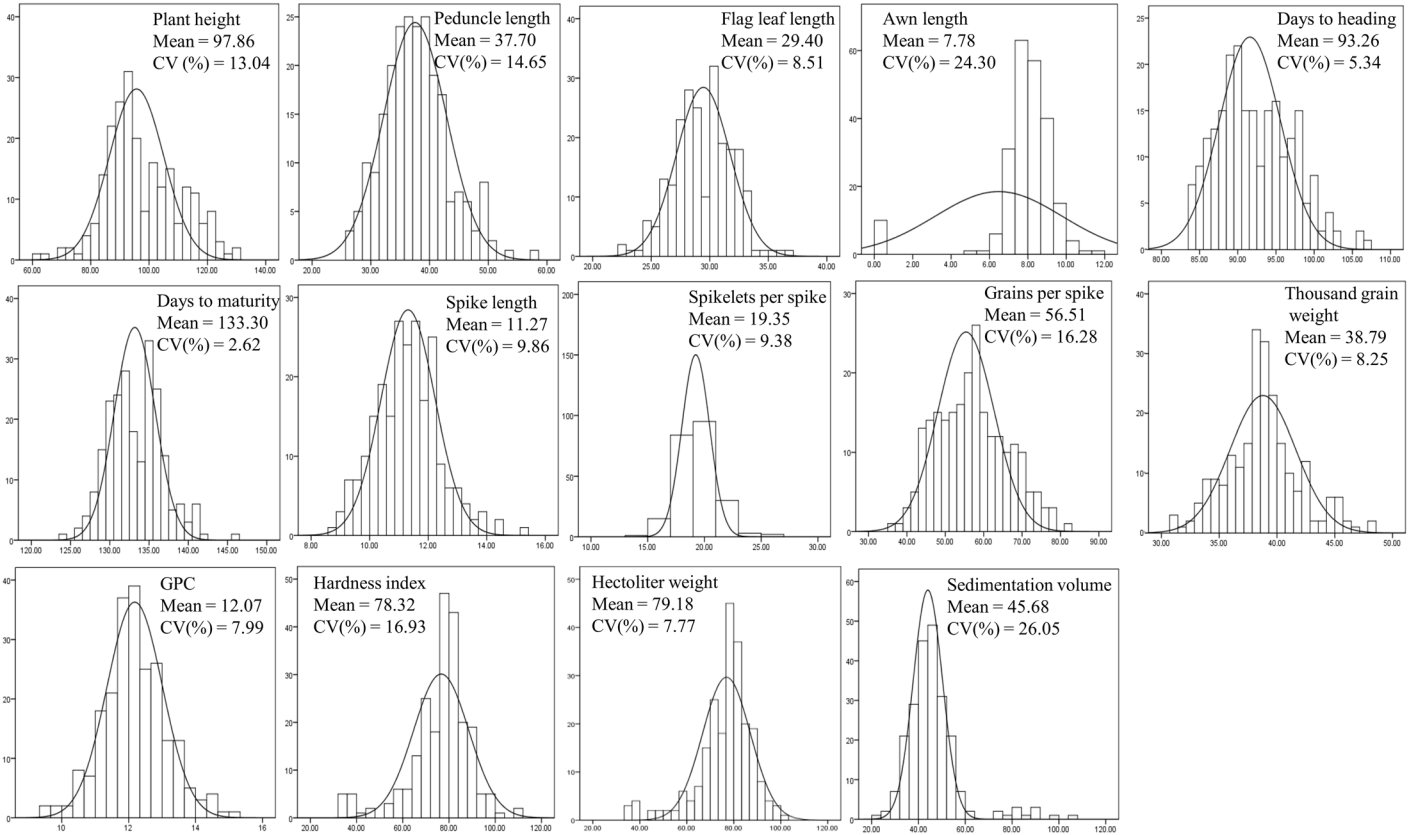
Frequency distribution of morphological, yield related and quality traits used for association mapping.

The data on distribution, mean values, and coefficient of variability (CV) for all the 14 traits involving 230 genotypes are presented in Fig 1. The extent of variability for the different traits suggested suitability of the association mapping panel for GWAS. Pearson’s correlation analyses revealed that 19 of the 91 possible pairs of traits (involving 14 traits) had significant (p-value≤ 0.05) and strong (r^2^ ≥ 0.25) correlations, making these pairs to be suitable for MTMM (S1 Table).

### Relationship between population structure and phenotypic data

The relationship of population structure with individual traits differed (reported by us earlier; for details, see Jaiswal et al. [22] and Mir et al. [11]), so that the traits were categorised in the following three groups on the basis of regression coefficient (r^2^) (i) 0% to 5% = poor relationship; (ii) 6% to 10% = moderate relationship; and (iii) >10% = strong relationship. Population structure showed poor relationship with HW (r^2^ = 4.3%) and DTH (r^2^ = 5.0%); moderate relationship with AL, DTM, GPC and TGW (r^2^ = 7.9% to 10.0%), and strong relationship with the remaining eight traits (SV, GS, SL, SKS, FL, HI, PL and PH; r^2^ = 11.1% to 34.0%) (Table 1).

**Table 1:** Relationships between the phenotypic traits and population structure computed using the mean values across environments. Probability (p-value) and r^2^ values (%) for phenotype–population structure relationship are based on the multiple regression analysis.

Traits	p-value	r^2^ (%)
PH	0.000	34.0^#^
PL	0.010	11.2^#^
FLL	0.001	14.4^#^
AL	0.035	9.5**
DTH	0.497	5.0*
DTM	0.109	7.9**
SL	0.000	16.0^#^
SKS	0.001	13.9^#^
GS	0.000	19.3^#^
TGW	0.024	10.0**
GPC	0.028	9.8**
HI	0.010	11.1^#^
HW	0.646	4.3*
SV	0.000	19.0^#^

* little relationship (r^2^ = 0.0% to 5.0%)

** moderate relationship (r^2^ = 6.0% to 10.0%)

^#^ high level relationship (r^2^ ≥ 11.0%)

### Model search for individual traits

**Fig 2:**
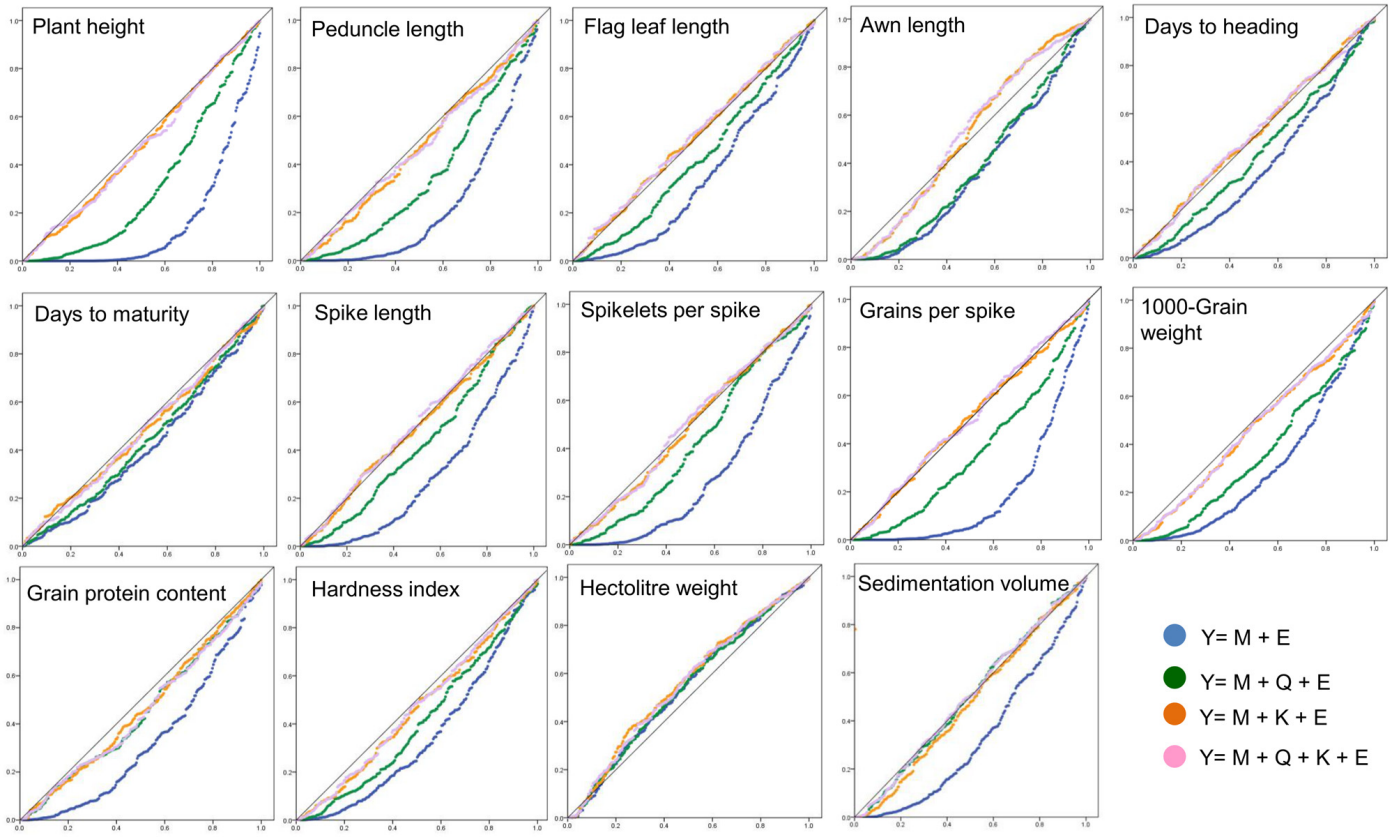
Plots of observed p-values (on y-axis) vs. expected p-values (on x-axis) for the 14 different traits using different association mapping models.

Values for mean square differences (MSD) for all the four models for each of the 14 traits along with the best fit models are summarised in S2 Table; corresponding Q-Q plots are given in Fig 2. Out of the four models that were tested, the naive model was not adequate for any of the 14 traits, Q model was best fit for HW only, K model was best fit for eight different traits (AL, GS, SL, SKS, DTH, FLL, GPC and PH) and Q+K model was best fit for the remaining five traits (SV, TGW, DTM, HI and PL). For individual traits, the MTAs were worked out using the best fit model.

### MTAs using SLST

**Fig 3:**
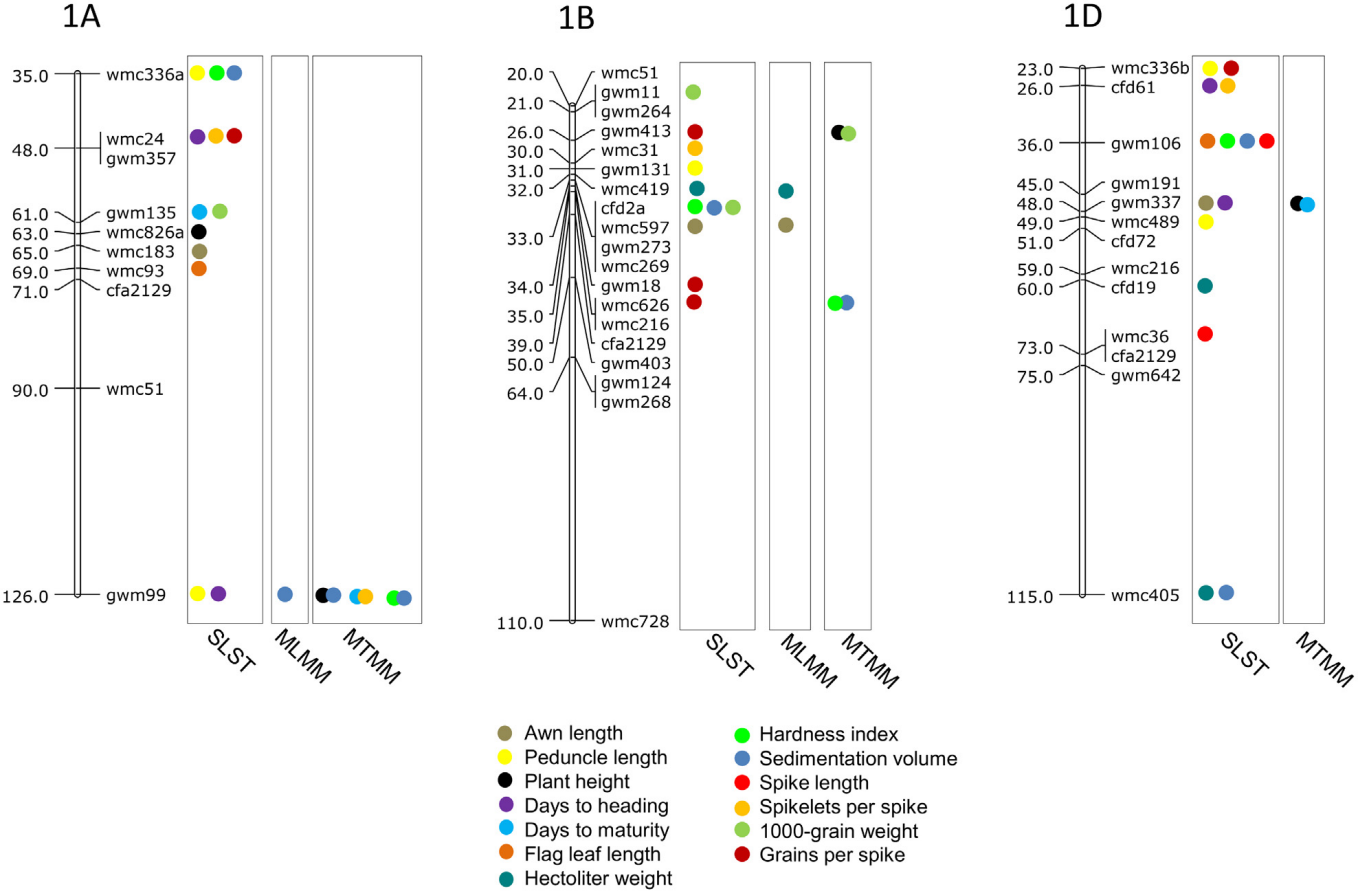
SSR genetic linkage maps of homoeologous group 1 chromosmes showing markers-trait associations (markers associated with different traits are shown by solid circles with different colours), markers are indicated to the right and map distances (cM) are indicated to the left of the vertical bar (based on the consensus linkage map of Somers et al. [76]).

**Fig 4:**
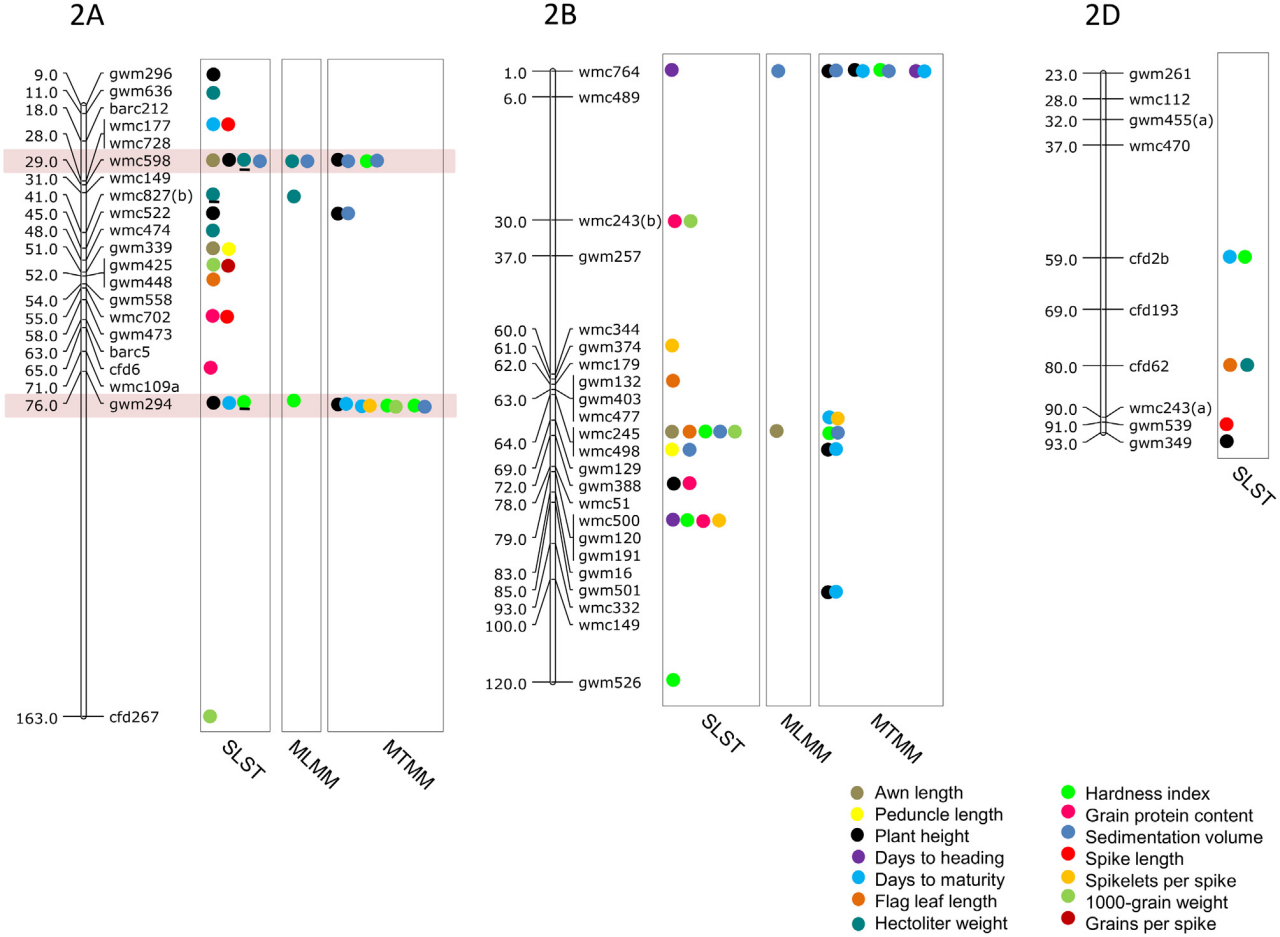
SSR genetic linkage maps of homoeologous group 2 chromosomes showing markers-trait associations (markers associated with different traits are shown by solid circles with different colours), black bar given below the solid circle represents that MTA qualified FDR criterion; markers are indicated to the right and map distances (cM) are indicated to the left of the vertical bar (based on the consensus linkage map of Somers et al. [76]). MTAs identified through all three approaches (SLST, MLMM and MTMM) are highlighted with pink.

**Fig 5:**
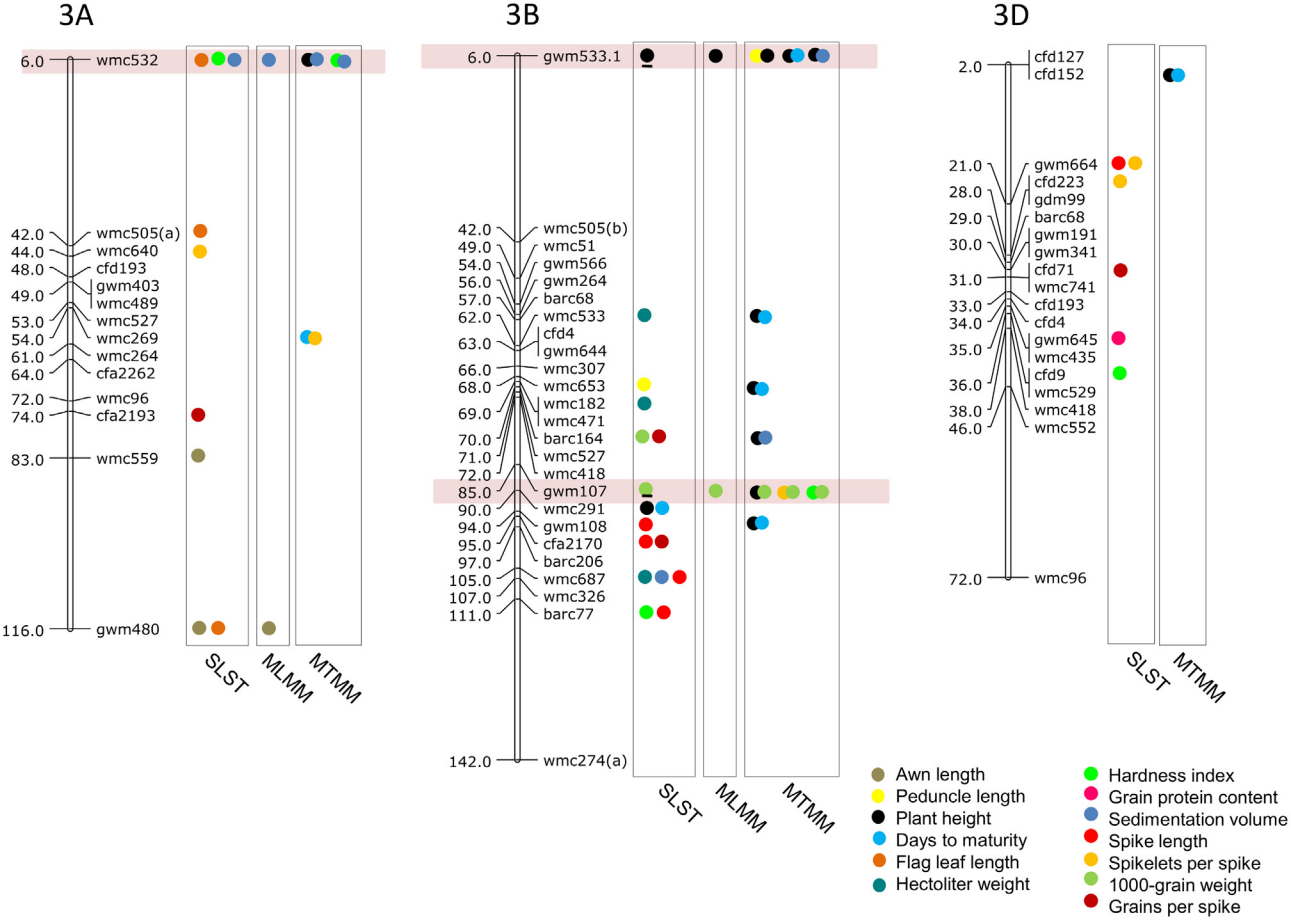
SSR genetic linkage maps of homoeologous group 3 chromosomes showing markers-trait associations (markers associated with different traits are shown by solid circles with different colours), black bar given below the solid circle represents that MTA qualified FDR criterion; markers are indicated to the right and map distances (cM) are indicated to the left of the vertical bar (based on the consensus linkage map of Somers et al. [76]. MTAs identified through all three approaches (SLST, MLMM and MTMM) are highlighted with pink.

**Fig 6:**
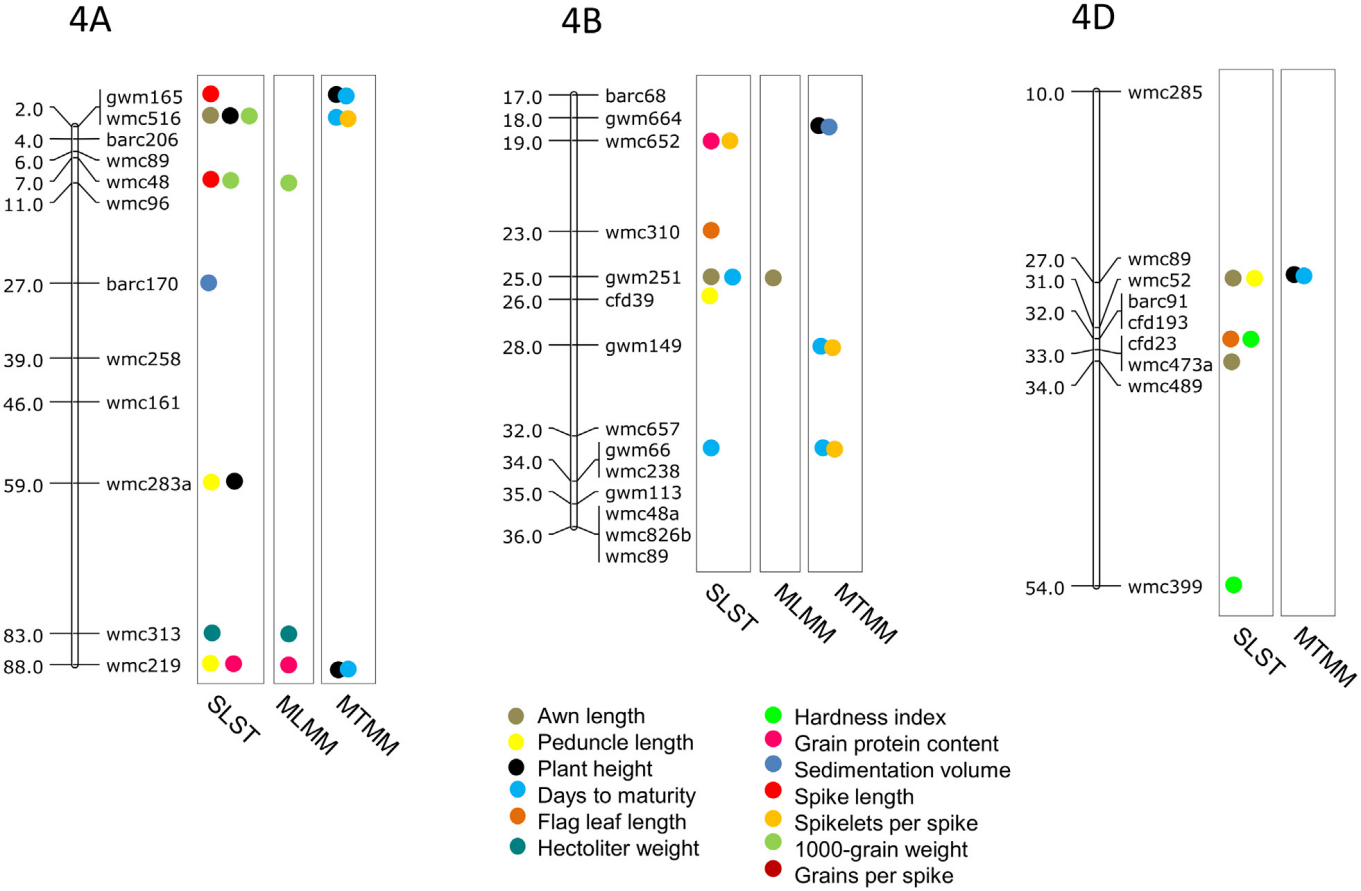
SSR genetic linkage maps of homoeologous group 4 chromosomes showing markers-trait associations (markers associated with different traits are shown by solid circles with different colours); markers are indicated to the right and map distances (cM) are indicated to the left of the vertical bar (based on the consensus linkage map of Somers et al. [76].

**Fig 7:**
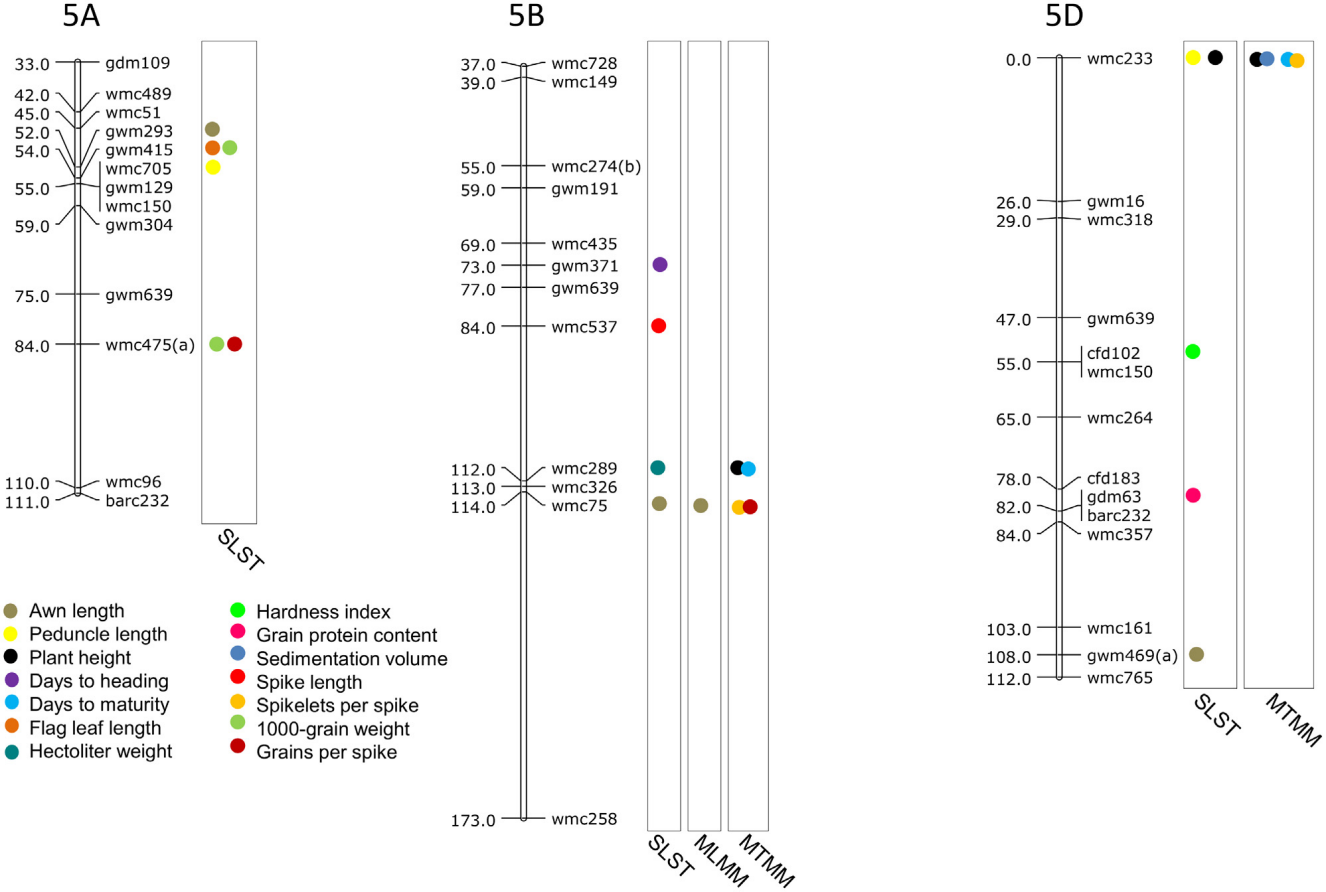
SSR genetic linkage maps of homoeologous group 5 chromosomes showing markers-trait associations (markers associated with different traits are shown by solid circles with different colours); markers are indicated to the right and map distances (cM) are indicated to the left of the vertical bar (based on the consensus linkage map of Somers et al. [76]).

**Fig 8:**
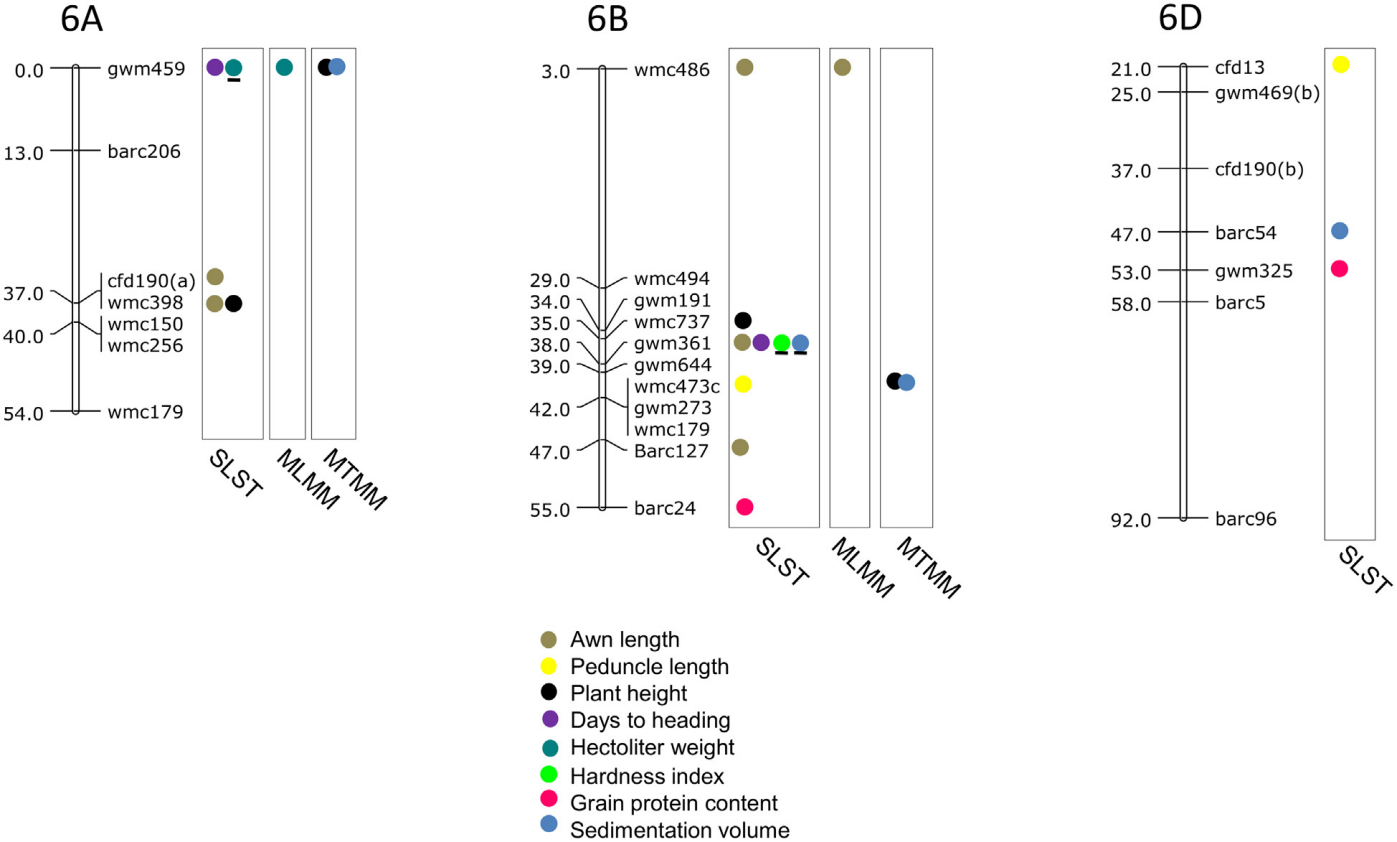
SSR genetic linkage maps of homoeologous group 6 chromsomes showing markers-trait associations (markers associated with different traits are shown by solid circles with different colours), black bar given below the solid circle represents that MTA qualified FDR criterion; markers are indicated to the right and map distances (cM) are indicated to the left of the vertical bar (based on the consensus linkage map of Somers et al. [76]).

**Fig 9:**
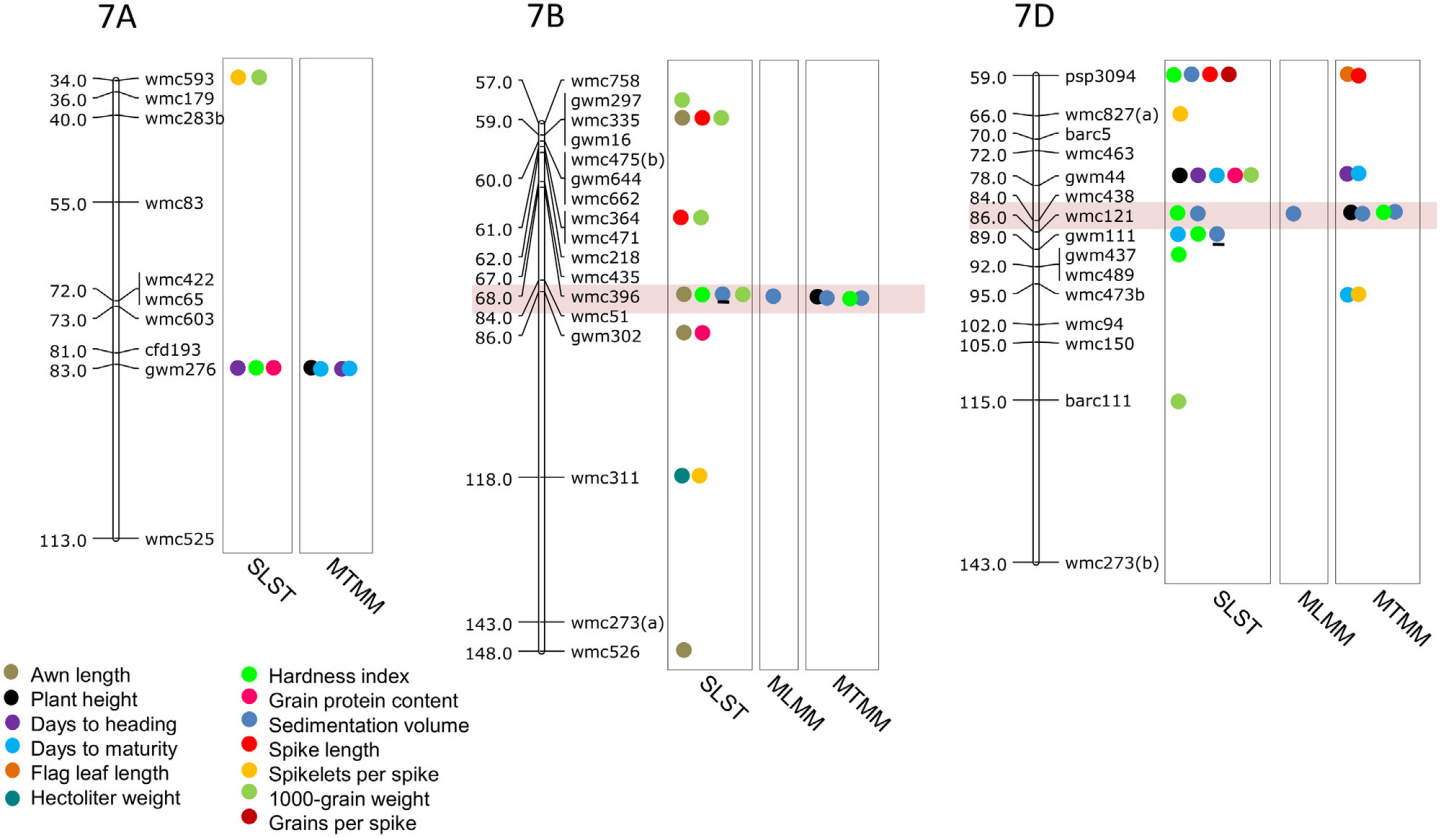
SSR genetic linkage maps of homoeologous group 7 chromosomes showing markers-trait associations (markers associated with different traits are shown by solid circles with different colours), black bar given below the solid circle represents that MTA qualified FDR criterion; markers are indicated to the right and map distances (cM) are indicated to the left of the vertical bar (based on the consensus linkage map of Somers et al. [76]). MTAs identified through all three approaches (SLST, MLMM and MTMM) are highlighted with pink.

Results of significant MTAs detected following SLST for each of the 14 traits are summarized in Table 2 and chromosomal location of SSRs involved in these MTAs are depicted in Figs 3–9. Altogether, 213 MTAs representing 203 QTLs involving 129 associated SSRs (spread over all the 21 chromosomes) were identified. Maximum number of SSRs (24) was associated with AL, and minimum number of SSRs (9) was associated with DTM (Table 2). Out of 129 associated SSRs, 72 SSRs were involved in single trait-specific MTAs and 57 SSRs were involved in multi-trait MTAs. Over all, only 10 MTAs involving 9 associated SSR markers (one SSR marker was shared with two traits) for five traits (PH, TGW, HI, HW and SV) qualified the FDR criteria (Table 3).

**Table 2:** Summary of the significant marker-trait associations (MTAs) for 14 traits detected using SLST. The results are referred to significant marker–trait associations on the basis of consistent marker-wise tests (P ≤ 0.05) carried out with best fit model of association mapping for individual trait.

Trait	No. of MTAs	p-value		R^2^ (%)		No. QTLs	No. of MTAs after FDR
		Min.	Max.	Min.	Max.		
PH	15	0.000	0.050	3.512	11.604	15	1
PL	16	0.003	0.046	1.794	9.874	16	0
FLL	12	0.004	0.050	2.848	8.294	11	0
AL	24	0.000	0.045	2.747	10.881	20	0
DTH	11	0.007	0.034	2.027	11.390	11	0
DTM	9	0.004	0.046	2.769	10.279	8	0
SL	16	0.002	0.047	1.980	7.720	11	0
SKS	12	0.002	0.049	2.002	12.346	11	0
GS	12	0.003	0.044	3.263	10.097	10	0
TGW	20	0.000	0.048	1.746	10.987	16	1
GPC	14	0.002	0.049	2.365	9.759	12	0
HI	21	0.000	0.050	1.935	13.805	19	2
HW	15	0.000	0.048	2.492	16.438	13	3
SV	16	0.000	0.044	3.116	15.530	14	3
Total	213	-	-	-	-	203	10

**Table 3:** List of the 10 most valuable (significant after FDR) MTAs involving 9 SSR markers that were detected during the present study using SLST approach and were also previously reported to affect related traits.

Locus	Chromosome	Genetic position (cM)	Associated trait	Previously identified loci affecting same or related traits*	
				Based on the sharing of common marker	Based on similarity in genetic position
wmc598	2A	29	HW	GWE [70]	wmc177(28.3 cM); TGW [47]
wmc827	2A	41	HW	-	cfa2201(41cM); HD. KPSM, TGW, GY [19]
gwm459	6A	0	HW	PL, KPSM [19]	gwm334 (2cM); TGW [73]
gwm533.1	3B	6	PH	GFR, TGW, GFD, FT [70]	-
gwm111	7D	89	SV	TGW [11]	-
gwm361	6B	38	SV, HI	TGW [71]	-
wmc396	7B	68	SV	PL, TW, GY [19]	-
gwm107	3B	85	TGW	-	barc115 (85.1cM); PH, KPSM, TGW [19]
gwm294	2A	76	HI	HD, PH, PL, KPSM, TGW, TW [19], SL, GPS [47], YLD, HD, SNP, SWP [72]	gwm312 (79.26cM); MTGW [51]

* GWE = grain weight/ear, PL = peduncle length, KPSM = kernel/square meter, GFR = grain filling rate, TGW = thousand grain weight, GFD = grain filling duration, FT = flowering time, TW = test weight, GY = grain yield, HD = heading date, SL = spike length, GPS = grain/spike, YLD = yield, SNP = spike number/plant, SWP = spike weight/plant, MTGW = mean thousand grain weight.

### MTAs using MLMM

Twenty two (22) MTAs (after FDR correction) for seven traits (PH, AL, TGW, GPC, HI, HW and SV) were identified following MLMM (Table 4). These MTAs involved 13 wheat chromosomes including 1A, 1B, 2A, 2B, 3A, 3B, 4A, 4B, 5B, 6A, 6B, 7B and 7D. Seven of the 22 MTAs, were common with those identified by SLST and qualified the FDR criteria. These seven MTAs included—gwm294 for HI; wmc419, wmc598 for SV; wmc827 for HW; gwm533.1 for PH; wmc396 for SV and; gwm107 for TGW. The remaining 15 MTAs largely figured among SLST MTAs, which did not qualify FDR.

**Table 4:** Significant marker-trait associations (MTAs) identified through multi-locus mixed model (MLMM) and qualified FDR criteria.

Trait	Marker	Chromosome	Position (cM)	p-value
AL	wmc597	1B	33	2.9 × 10^−4^
	wmc245	2B	64	4.3 × 10^−5^
	gwm480	3A	116	1.8 × 10^−4^
	gwm251	4B	25	3.3 × 10^−6^
	wmc75	5B	114	2.5 × 10^−6^
	wmc486	6B	3	2.2 × 10^−5^
GPC	wmc219	4A	88	2.4 × 10^−5^
HI	gwm294*^,^^$^	2A	76	3.1 × 10^−6^
HW	wmc419*	1B	32	3.6 × 10^−6^
	wmc598*	2A	29	1.6 × 10^−5^
	wmc827*	2A	41	5.9 × 10^−6^
	wmc313	4A	83	2.7 × 10^−5^
	gwm459	6A	0	1.0 × 10^−8^
PH	gwm533.1*^,^^$^	3B	6	1.9 × 10^−5^
SV	gwm99^$^	1A	126	2.2 × 10^−4^
	wmc598^$^	2A	29	2.9 × 10^−4^
	wmc764^$^	2B	1	8.8 × 10^−8^
	wmc532^$^	3A	6	3.6 × 10^−7^
	wmc396*^,^^$^	7B	68	2.1 × 10^−11^
	wmc121^$^	7D	86	2.5 × 10^−4^
TGW	gwm107*^,^^$^	3B	85	1.0 × 10^−5^
	wmc48	4A	7	1.0 × 10^−4^

*^,^^$^ MTAs also identified through single locus-single trait (qualified FDR) and MTMM analysis, respectively.

### MTAs using MTMM

MTMM analyses allowed identification of 58 MTAs (after FDR correction) representing 29 QTLs) for 11 pairs of correlated traits. These 58 MTAs involved 18 of the 21 wheat chromosomes with the exception of 2D, 5A and 6D. As many as 32 MTAs were identified using full test, 43 were identified using interaction test and 9 were identified using common marker test (a number of MTAs were identified using more than one method). Nine pleiotropic QTLs for the following three pairs of correlated traits were identified using common marker test: PH-TGW (1 MTA), PH-SV (5 MTAs) and DTH-DTM (3 MTAs). Out of 58 MTAs, eight MTAs were common with SLST (which qualified FDR), and nine MTAs were common with MLMM analyses (Tables 4 and 5). The number of MTAs for individual pairs of correlated traits ranged from 1 to 14. There were two pairs of correlated traits, namely PH-DTM and PH-SV, for each of which a maximum of 14 MTAs were available; in contrast, for each of following four pairs of correlated traits, a solitary MTA was available: PH-PL, FLL-SL, SKS-GS and SKS-TGW).

**Table 5:** Significant marker-trait associations identified through multi-trait mixed model (MTMM) and qualified FDR criteria. MTMM was performed for pair of correlated traits (for significant and high correlation; p value ≤0.05, r^2^ value ≥ 2.5).

Trait combination	Marker	Chromosome	p-value		
			Full test	Interaction	Common
PH / PL	gwm533.1*	3B	1.4 × 10^−5^	-	-
PH / DTM	gwm337	1D	-	1.5 × 10^−4^	-
	gwm294	2A	7.3 × 10^−5^	-	-
	wmc498	2B	6.3 × 10^−7^	1.7 × 10^−7^	-
	gwm501	2B	-	1.1 × 10^−4^	-
	gwm533.1*	3B	1.1 × 10^−4^	-	-
	wmc653	3B	7.8 × 10^−5^	1.8 × 10^−5^	-
	gwm108 (b)	3B	-	8.7 × 10^−5^	-
	wmc533.1	3B	-	2.3 × 10^−4^	-
	cfd152	3D	2.95 × 10^−5^	5.3 × 10^−6^	-
	wmc219	4A	-	2.2 × 10^−4^	-
	gwm165	4A	-	5.1 × 10^−4^	-
	wmc52	4D	-	2.7 × 10^−4^	-
	wmc289	5B	-	3.7 × 10^−4^	-
	gwm276	7A	1.1 × 10^−4^	4.6 × 10^−5^	-
PH / TGW	gwm413	1B	-	6.2 × 10^−5^	-
	gwm107*	3B	1.4 × 10^−5^	-	6.4 × 10^−5^
PH / SV	gwm99	1A	2.4 × 10^−4^	-	-
	wmc598	2A	2.3 × 10^−5^	1.4 × 10^−5^	-
	wmc522	2A	2.4 × 10^−4^	4.5 × 10^−5^	-
	wmc764	2B	5.6 × 10^−8^	6.6 × 10^−5^	2.9 × 10^−5^
	wmc532	3A	1.3 × 10^−7^	3.4 × 10^−4^	1.4 × 10^−5^
	gwm533.1*	3B	1.5 × 10^−4^	-	2.7 × 10^−5^
	wmc291	3B	3.8 × 10^−4^	1.3 × 10^−4^	-
	barc164	3B	-	4.3 × 10^−4^	-
	gwm664	4B	-	4.7 × 10^−4^	-
	wmc233	5D	-	-	1.9 × 10^−4^
	wmc256	6A	4.1 × 10^−4^	-	-
	wmc473	6B	-	1.8 × 10^−4^	-
	wmc396*	7B	1.3 × 10^−11^	2.7 × 10^−8^	1.2 × 10^−5^
	wmc121	7D	4.0 × 10^−4^	-	-
FLL / SL	psp3094	7D	1.6 × 10^−6^	1.3 × 10^−6^	-
DTH / DTM	wmc764	2B	-	-	5.4 × 10^−5^
	gwm276	7A	-	-	7.9 × 10^−5^
	gwm44	7D	-	-	1.9 × 10^−4^
DTM / SKS	gwm99	1A	-	5.3 × 10^−5^	-
	gwm294	2A	-	1.6 × 10^−4^	-
	wmc477	2B	-	3.6 × 10^−4^	-
	wmc764	2B	-	5.0 × 10^−4^	-
	wmc269	3A	-	2.0 × 10^−4^	-
	wmc516	4A	1.6 × 10^−5^	2.8 × 10^−6^	-
	gwm149	4B	-	2.1 × 10^−4^	-
	gwm66	4B	-	3.6 × 10^−4^	-
	wmc233	5D	8.2 × 10^−5^	1.6 × 10^−5^	-
	wmc473 b	7D	-	2.4 × 10^−4^	-
SKS / GS	wmc75	5B	-	5.6 × 10^−5^	-
SKS / TGW	gwm107	3B	2.7 × 10^−5^	-	-
TGW / HI	gwm294	2A	1.3 × 10^−6^	1.5 × 10^−6^	-
	gwm107*	3B	7.9 × 10^−5^	-	-
HI / SV	gwm99	1A	1.5 × 10^−4^	8.7 × 10^−5^	-
	wmc626	1B	-	2.8 × 10^−4^	-
	gwm294*	2A	2.1 × 10^−5^	3.7 × 10^−5^	-
	wmc598	2A	3.2 × 10^−4^	-	-
	wmc764	2B	6.0 × 10^−8^	1.4 × 10^−6^	-
	wmc245	2B	1.6 × 10^−4^	6.0 × 10^−5^	-
	wmc532	3A	1.9 × 10^−8^	1.5 × 10^−8^	-
	wmc396*	7B	2.4 × 10^−12^	2.9 × 10^−10^	-
	wmc121	7D	3.5 × 10^−4^	1.8 × 10^−4^	-

* MTAs also identified through single locus analysis(qualified FDR).

### Main effect QTLs involved in epistatic interactions

**Fig 10:**
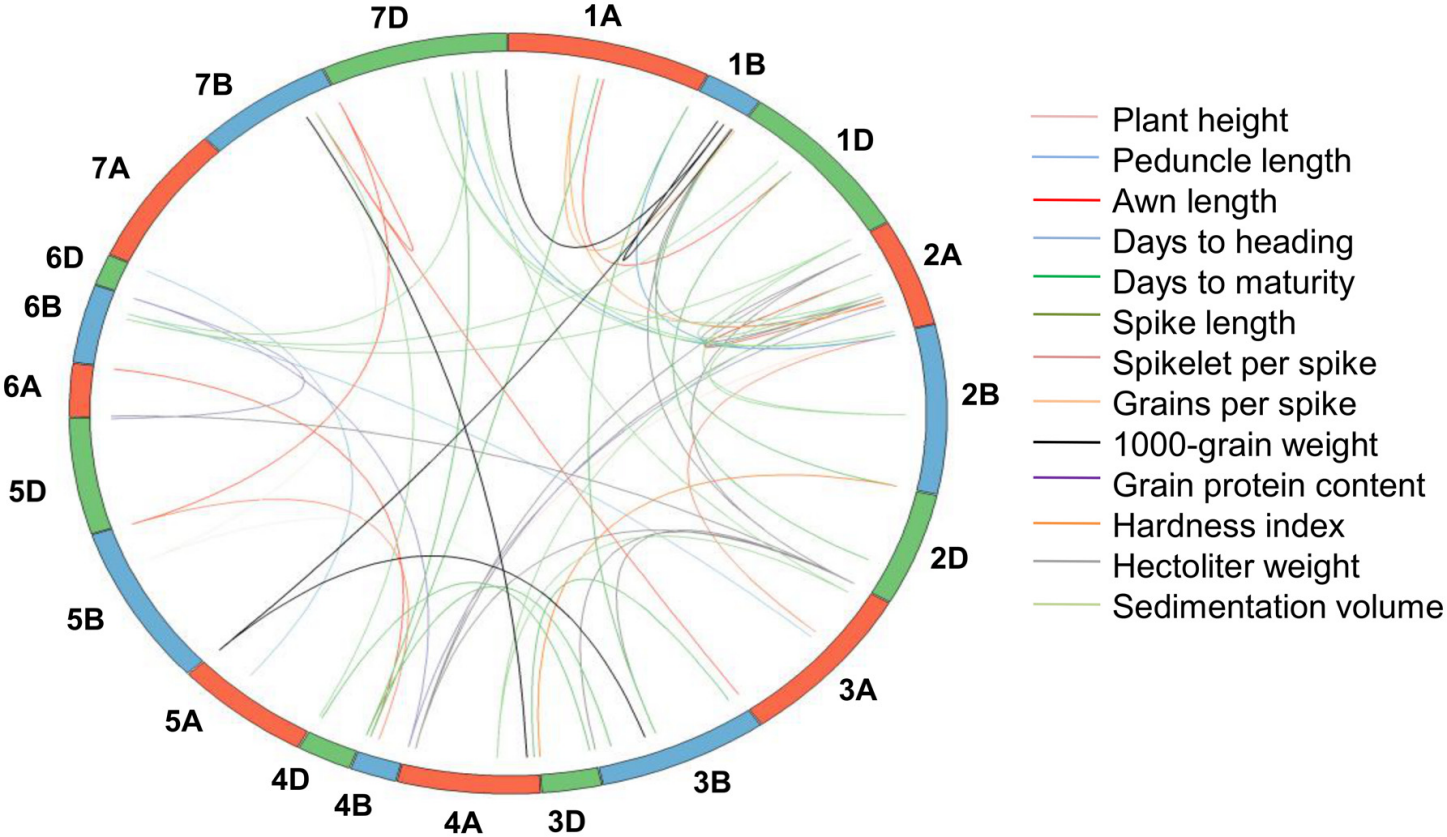
Epistatic interactions using main effect markers (identified by SLST, MLMM and MTMM) for 13 traits. Cut off p-value for significant interaction is ≤ 0.001. Interactions for different traits are represented by different coloured lines.

As many as 63 epistatic interactions were identified for 13 (out of 14) traits (Table 6), FLL, being the only exception. Markers involved in the above mentioned 63 epistatic interactions were spread over all the wheat chromosomes except 7A (Fig 10). A maximum of 13 interactions were observed for SV, while a minimum of one interaction was detected for PH.

**Table 6:** Epistatic interactions using main effect markers (identified by SLST, MLMM and MTMM) for 13 traits along with their p-value.

Trait	Marker	Chromosme	Position	Marker	Chromosome	Position (cM)	p value (<.001)
AL	wmc183	1A	65	gwm337	1D	48	0.00093
	wmc598	2A	29	gwm339	2A	51	0.00045
	gwm480	3A	116	wmc396	7B	68	0.00015
	gwm251	4B	25	cfd190(a)	6A	37	0.00054
	wmc75	5B	114	gwm302	7B	86	0.00033
	wmc396	7B	68	gwm302	7B	86	0.00052
DTH	gwm99	1A	126	wmc764	2B	1	0.00149
	wmc764	2B	1	gwm44	7D	78	0.00120
DTM	gwm99	1A	126	wmc533	3B	62	0.00029
	gwm135	1A	61	gwm251	4B	25	0.00049
	wmc269	1B	33	gwm526	2B	120	0.00122
	gwm337	1D	48	cfd2b	2D	59	0.00041
	gwm294	2A	76	wmc473b	7D	95	0.00096
	gwm533.1	3B	6	wmc516	4A	2	0.00090
	gwm108(b)	3B	94	gwm149	4B	28	0.00125
	gwm533.1	3B	6	gwm165	4A	2	0.00143
	cfd152	3D	2	wmc52	4D	31	0.00104
	gwm149	4B	28	gwm44	7D	78	0.00045
GPC	wmc702	2A	55	wmc219	4A	88	0.00100
	wmc219	4A	88	barc24	6B	55	0.00058
	gdm63(b)	5D	82	barc24	6B	55	0.00149
HI	gwm106	1D	36	gwm361	6B	38	0.00131
	gwm294	2A	76	psp3094	7D	59	0.00052
	gwm526	2B	120	cfd9	3D	36	0.00117
HW	wmc419	1B	32	wmc474	2A	48	0.00024
	wmc419	1B	32	cfd62	2D	80	0.00123
	gwm636	2A	11	wmc313	4A	83	0.00020
	gwm636	2A	11	wmc474	2A	48	0.00065
	wmc598	2A	29	wmc474	2A	48	0.00093
	wmc474	2A	48	wmc313	4A	83	0.00105
	gwm636	2A	11	cfd62	2D	80	0.00150
	cfd62	2D	80	wmc533	3B	62	0.00020
	cfd62	2D	80	wmc687	3B	105	0.00047
	cfd62	2D	80	wmc313	4A	83	0.00073
	cfd62	2D	80	gwm459	6A	0	0.00114
PH	gwm294	2A	76	wmc219	4A	88	0.00011
PL	wmc489	3A	49	wmc473c	6B	42	0.00126
	wmc705	5A	55	cfd13	6D	21	0.00043
SKS	gwm99	1A	126	wmc764	2B	1	0.00149
	wmc764	2B	1	wmc640	3A	44	0.00078
	gwm664	4B	19	wmc75	5B	114	0.00131
SL	cfa2170(b)	3B	95	wmc537	5B	84	0.00132
	wmc537	5B	84	wmc364(a)	7B	61	0.00140
GS	wmc24	1A	48	wmc626	1B	35	0.00071
	wmc24	1A	48	gwm425(b)	2A	52	0.00137
SV	wmc405	1D	115	wmc522	2A	45	0.00032
	wmc405	1D	115	gwm361	6B	38	0.00107
	wmc405	1D	115	wmc245	2B	64	0.00120
	gwm106	1D	36	gwm361	6B	38	0.00131
	gwm294	2A	76	psp3094	7D	59	0.00052
	wmc598	2A	29	wmc498	2B	64	0.00066
	wmc522	2A	45	barc170	4A	27	0.00080
	wmc522	2A	45	psp3094	7D	59	0.00095
	gwm294	2A	76	wmc473b	7D	95	0.00096
	wmc532	3A	6	barc170	4A	27	0.00053
	wmc532	3A	6	wmc473b	7D	95	0.00145
	wmc473	4D	33	wmc396	7B	68	0.00073
	wmc473c	6B	42	wmc121	7D	86	0.00124
TGW	gwm413	1B	26	wmc475(a)	5A	84	0.00038
	gwm11	1B	21	cfd2a	1B	33	0.00046
	gwm413	1B	26	barc111	7D	115	0.00086
	barc164	3B	70	wmc475(a)	5A	84	0.00093
	wmc48	4A	7	wmc364(a)	7B	61	0.00109

### Identification of important rare alleles and rare variants

During genotyping of 230 cultivars, 250 SSRs exhibited a total of 1124 alleles; 316 of these alleles (representing 165 SSRs) were rare alleles, each with a frequency of 5% or less. The genotypes carrying these rare alleles are described as rare variants for individual traits. For individual SSRs, these rare alleles ranged from 1 to 9 with a mean of 1.26 alleles per SSR. For individual traits, these rare alleles were carried by 1 to 11 rare variants (since same rare allele may be carried by more than one rare variants). The rare variants were examined for each individual trait to identify the specific rare variants, which carried a desirable state of the phenotype for each trait. Such important and desirable rare variants carried only 78 of the 316 rare alleles belonging to 55 SSRs (S3 Table); these 78 rare alleles and the corresponding rare variants, each with desirable state of one or more individual traits were considered important and need attention (see Discussion). Rare variants with desirable state of individual traits were available only for 10 of the 14 traits examined (for 4 traits, namely DTM, SV, PL and SL, no desirable and important rare variants were available); the number of rare alleles carried by important/desirable rare variants varied for individual traits and ranged from 2 (for HW) to 17 (for DTH).

### Important MTAs, QTL alleles and genotypes

Using the criteria mentioned earlier, 56 MTAs involving 38 SSRs for 11 traits were considered important; some of these SSRs were involved in more than one traits (S4 Table); for the remaining three traits (FLL, PL and SKS), the available MTAs neither qualified for FDR, nor were these reported in earlier literature; these were, therefore ignored. MTAs for each of the 11 traits are listed in Table 7.

**Table 7:** SSRs involved in important MTAs identified through different approaches or their combinations.

S. No.	Trait	SSRs involved in important MTAs
1.	AL	gwm251^d^, gwm480^d^, wmc245^d^, wmc486^d^, wmc597^d^, wmc75^d^
2.	DTH	gwm276^e^, gwm44^e^, wmc764^e^
3.	DTM	gwm276^c^, gwm294^e^, gwm44^e^, gwm66^e^, wmc764^c^
4.	GPC	wmc219^d^
5.	GS	gwm413^b^, wmc24^b^
6.	HI	gwm294^a^^,^^g^, wmc121^e^, wmc245^e^, wmc532^e^, gwm361^a^
7.	HW	gwm459^a^^,^^d^, wmc313^d^, wmc419^d^, wmc598^a^^,^^d^, wmc827^a^^,^^d^
8.	PH	gwm107^c^, gwm294^e^, gwm296^b^, gwm349^b^, gwm533.1^a^^,^^g^, wmc396^c^, wmc522^e^, wmc532^c^, wmc598^e^, wmc764^c^
9.	SL	psp3094^e^, wmc702^b^
10.	SV	gwm111^a^, gwm533.1^c^, gwm99 ^f^, wmc121^g^, wmc233^c^, wmc245^e^, wmc396^a^^,^^g^, wmc532^g^, wmc598^g^, wmc764^f^, gwm361^a^
11.	TGW	barc164^b^, gwm107^a^^,^^g^, gwm11^b^, wmc48^e^, wmc516^b^, wmc593^b^

^a^ SLST (qualified FDR),

^b^ SLST only (did not qualified FDR but reported in earlier studies),

^c^ MTMM only,

^d^ SLST+MLMM,

^e^ SLST+MTMM,

^f^ MLMM+MTMM,

^g^ SLST+MLMM+MTMM

The 38 desirable QTLs (associated with 38 associated SSRs) included 12 main effect QTLs that were not involved in epistatic interactions; the remaining 26 QTLs were also involved in epistatic interactions. A set of 17 superior genotypes carrying desirable alleles for important QTLs for 11 traits were also identified (Table 8). Some of these genotypes carried superior alleles for only one trait. Therefore, other genotypes carrying superior alleles for two traits may be preferred over the genotypes carrying superior alleles for a single trait. Eventually, from above 17 genotypes, only the following eight genotypes were selected, which carried superior alleles for either two traits [WH542 (HI and PH), Sharbati Sonora (DTH and DTM)]; or had most desirable trait value in case, where superior allele for a solitary trait was available [NI345 (SV), NI179 (TGW), A90 (HW), HW1085 (GS), HYB 11 (GPC), and DWR39 (Pragati) (AL)], see Table 8.

**Table 8:** A summary of the 17 superior genotypes and markers with desirable alleles associated with respective traits. Number given after bar (-) represents desirable allele size (in bp). Two markers, separated by “/”, are involved in epistatic interaction; and among these pairs of interacting markers, the first marker was identified by either one, two, or by all the three approaches (SLST, MLMM, MTMM). Eight genotypes considered more important are highlighted with bold.

S. No.	Genotype	Trait	Trait value	Desirable marker allele/combinations of alleles
**1**	**WH542**	HI	102.4	gwm294-83^a^^,^^f^
		PH	82cm	gwm533.1–122^a^^,^^f^
**2**	**SHARBATI SONORA**	DTH	84	gwm276-73^e^, gwm44-182^e^/wmc764-193, wmc764-193^e^/gwm99-124
		DTM	126	gwm294-100^e^/wmc473-141, gwm44-182^e^/gwm149, gwm66^e^, gwm276-73^c^, wmc764-193^c^
3	K816	PH	72cm	Wmc598-null^e^
4	LAL BAHADUR	PH	62cm	gwm296-124^b^, gwm349-null^b^, gwm107-170c, wmc764-193^c^, wmc532-195^c^, wmc396-145^c^, wmc522-224 ^e^, gwm294-105^e^/ wmc219-126
**5**	**DWR39**	AL	11.59mm	wmc597-191^d^, wmc245-154^d^, gwm480-334^d^/wmc396-145, gwm251-170^d^/cfda-218, wmc75-234^d^/gwm302
6	UP2425	SL	13.58cm	wmc702-229^b^
7	VL616	SL	14.55cm	psp3094-136^e^
8	KALYAN SONA	GS	78	gwm413-77^b^, wmc24-152 ^b^
**9**	**HW1085**	GS	81	gwm425-133,wmc621-151 (interacting loci of wmc24)
10	KENPHAD	TGW	48.2g	gwm11-218^b^/ cfd2–171
**11**	**NI179**	TGW	48.5g	gwm107-192^a^^,^^f^, wmc48-194^e^/wmc364-192, barc164-263^b^, wmc593-146^b^, wmc516-137^b^
**12**	**HYB11**	GPC	15.32%	wmc219-152^d^/wmc702-234/barc24-110
13	WG357	HI	110.6	gwm361-134^a^, wmc245-145^e^, wmc532-195^e^, wmc121-333^e^
**14**	**A90**	HW	95.1g	wmc598-null^a^^,^^d^, wmc827-237^a^^,^^d^, gwm459-122^a^^,^^d^, wmc419-160^d^, wmc313-176^d^
**15**	**NI345**	SV	106.4ml	wmc598-161^f^/ wmc498-164, wmc532-195^f^/barc170-174, wmc396-167^a^^,^^f^, gwm361-134^a^, gwm111-null^a^, gwm533.1–161^c^, wmc233-190^c^
16	NP120	SV	96.5ml	wmc121-328^f^/wmc473-212
17	NP721	SV	89.9ml	gwm99-103^e^, wmc764-193^e^, wmc245-154^e^/wmc405-115

^a^ SLST (qualified FDR),

^b^ SLST only (did not qualified FDR but reported in earlier studies),

^c^ MTMM only,

^d^ SLST+MLMM,

^e^ SLST+MTMM,

^f^ SLST+MLMM+MTMM

## Discussion

The present association mapping study (GWAS) has the following important/novel features of interest. Firstly, it addresses the problem of trait-related population structure, secondly it provides improvement upon SLST analysis through the use of MLMM and MTMM, thirdly it includes identification of epistatic interactions, which are seldom included in GWAS, and finally effort has been made to highlight the problem of rare alleles and rare variants, which is currently one of the most widely debated issues in GWAS. It is known that during GWAS, confounding arises due to population structure, particularly if it is correlated with the trait under study [22]. In the present study, model selection allowed us to address this problem of trait-related population structure. It has been documented that population structure, if related with the trait of interest may lead to erroneous conclusions as shown in case of *Dwarf4* gene of maize [38–39]. Keeping this in mind, model search was made, so that, models used in the present study differed for different individual traits (Q-model, K-model or Q + K model; for details see materials and methods), depending on whether or not the population structure had a relationship with the traits under study; only the most appropriate model was used for each of the 14 individual traits [40–41]. Thus, the use of appropriate models showing best fit provided higher level of confidence in our association mapping results. In the past, most of the association mapping studies in wheat, with few exceptions [41–42], arbitrarily used either the Q-model, or the K-model or the Q + K-model without first examining, the best fit model for each trait, thus leading to results with low level of confidence.

FDR corrections were also used in the present study. It may be recalled, that during SLST analysis, only nine markers involved in 10 MTAs, out of 213 MTAs, could qualify after FDR corrections; all these 10 MTAs fall within the genomic regions earlier reported to be associated with the corresponding or related traits in wheat, placing higher level of confidence in these MTAs (Table 3). However, we recognize that FDR correction is actually a trade-off (between identification of MTAs with higher level of confidence and the inflation in the number of false negatives), so that some genuine associations escape detection as false negatives [23, 43]. Therefore, we examined further the remaining 203 MTAs (after excluding the above 10 MTAs), which did not qualify FDR criterion. On comparison with already reported MTAs, we found that nine of the 203 MTAs (that did not qualify FDR criteria) for four different traits (TGW, GS, SL and PH) were reported by one or more of the earlier QTL mapping studies (Table 9). These nine MTAs involved the following: gwm11, barc164, wmc593, wmc516 for TGW; wmc24, gwm413 for GS; wmc702 for SL; and gwm296, gwm349 for PH [11, 44–51]. These examples illustrate that the MTAs, which fail FDR correction need not be ignored and should be further examined for their validation through linkage mapping using suitably designed biparental mapping populations.

**Table 9:** List of significant MTAs involving nine SSR markers which did not qualify FDR criteria but reported in earlier studies.

Trait	Markers	References
TGW	gwm11, barc164, wmc593, wmc516	[11, 44–46, 51, 74–75]
GS	wmc24, gwm413	[48–50]
SL	wmc702	[47]
PH	gwm296, gwm349	[48]

The problem of genetic background affecting the detection of QTL was addressed during the present study through the use of MLMM [28], since each of the 14 traits used in the present study are quantitative in nature [3, 5, 14, 52], so that the power of detection of QTLs is adversely affected by genetic background. Using MLMM approach, 15 additional MTAs were detected, which were not really unique, but occurred among those SLST MTAs, which did not qualify FDR, once again suggesting that MTAs, which did not qualify FDR, should not be ignored, and need to be further examined.

The use of MTMM during the present study also allowed identification of 9 MTAs involving QTL that are associated with three pairs of correlated traits (out of 19 pairs of correlated traits examined). This suggested that the remaining correlations were either due to environmental effect or due to LD rather than due to pleiotropy/linkage. These 9 MTAs may also prove useful for simultaneous improvement of correlated traits. Further, out of 9 SSRs involved in above mentioned 9 MTAs, only one SSR (gwm44) was found to be associated with corresponding traits (DTH and DTM) in SLST analysis. The remaining MTAs involving 8 SSRs could not be detected using SLST and MLMM suggesting higher power of AM through MTMM. However, we speculate that power of AM may be further increased by using combined multi-locus multi-trait analysis.

MTMM, however, also has certain limitations. For instance, unlike joint analysis of QTL Cartographer, which examines more than two traits simultaneously, MTMM allows analysis of only pairs of correlated traits, so that pleiotropic QTL controlling more than two traits cannot be identified, although correlation studies do suggest that more than two traits may be correlated with each other in all possible combinations (S1 Table). We recognize that MTMM can be extended from pairs of traits to multi-trait analysis to elucidate functional relationship among several-traits; such multi-trait association mapping studies have recently been conducted in beef cattle [53] and human [54]; more such studies in plants are likely to be conducted in future.

None of the three approaches (SLST, MLMM, MTMM) discussed above deals with epistatic interactions during routine analysis. Estimation of epistasis, however, is important to understand genetic architecture [55–56] and a lack of such knowledge may result in under-utilization of genomic information for crop improvement [57]. However, the epistatic interactions have been sparingly examined during GWAS, despite their importance both for understanding the genetic architecture of the agronomic traits and their exploitation in trait improvement through MAS [10, 58–62]. The role of epistasis in wheat cannot be overemphasized as already demonstrated in case of flowering time [10, 62] and stem rust resistance [59–60]. In fact, substantially higher (93%) total genotypic variance for flowering time could be explained when epistatic interactions were taken into account, while main effects alone explained only 46% of genotypic variance [10]. During the present study, an examination of epistatic interactions among the main effect loci detected following SLST, MLMM and MTMM approaches allowed detection of 63 epistatic interactions for 13 traits (Table 6), suggesting that the epistasis plays an important role in the genetic control of these traits. Thus, the pairs of loci involved in epistatic interactions are equally important and may be exploited for crop improvement after due validation. Also, the possibility of interactions among loci other than main effect loci and the higher order of interactions involving more than two loci (e.g. QTL x QTL x QTL) cannot be ignored, although such interactions could not be studied during the present study. However, epistatic QTL without main effect using QTLNetwork for interval mapping [63], and higher-order epistatic interactions using Bayesian High-order Interaction Toolkit (BHIT) have been successfully used in the past [64].

Important MTAs and QTLs were also examined for their utility in MAS. Since genes/QTLs need to be transferred/pyramided in different genetic backgrounds using MAS, one should identify important gene/QTLs, which are context- independent and whose expression is not affected by change in genetic background. We identified 56 important MTAs involving 38 SSRs (12 SSRs associated with more than one trait) for 11 agronomic traits excluding PL, SKS and FLL; several of these MTAs were also reported in earlier studies (see, S4 Table), suggesting their utility in MAS for wheat improvement. Notably, 26 loci of the above 38 important loci for 10 of the 11 traits (excluding PH) were also involved in epistatic interactions (S4 Table). Thus, the pairs of loci involved in epistatic interactions are equally important and may be exploited for crop improvement after due validation.

In summary, based on the present study, we conclude that the following classes of MTAs, which are often ignored, may be equally useful for MAS: (i) MTAs, which do not qualify FDR correction in SLST analysis but are reported in earlier studies; (ii) MTAs that are context-independent, so that an introgression of desirable traits into unrelated genetic backgrounds may be successfully achieved; (iii) MTAs involving pleiotropic QTL/genes that can improve more than one desirable traits simultaneously, and (iv) MTAs involved in epistatic interactions, so that additional desirable genetic variation due to epistatic interactions may be exploited. In view of this, following eight genotypes which carried superior alleles for one or more traits were identified for future wheat breeding programmes: WH542 (HI and PH), Sharbati Sonora (DTH and DTM), NI345 (SV), NI179 (TGW), A90 (HW), HW1085 (GS), HYB 11 (GPC), and DWR39 (Pragati)(AL). These wheat genotypes were released in India for commercial cultivation during a period of 79 years spread from 1919 to 1998 and thus constitute breeding material not in current use. Therefore, we propose that the genetic variability available in these eight genotypes may be exploited by involving these genotypes in crosses to derive one or more multi-parental populations (MPP), each segregating for majority of QTLs. Such MPP may be subjected to molecular marker-assisted recurrent selection (MARS). This should allow selection of genotypes with superior alleles for main effect as well as epistatic QTL. Alternatively, desirable alleles available in the above eight genotypes may be introgressed and rapidly pyramided into the currently grown wheat cultivars to develop superior wheat genotype following pseudo-backcrossing as done in rice recently [65]. These improved genotype(s) may result into cultivars with improved agronomic performance and grain quality or may constitute important genetic resource for future wheat breeding programmes.

Another issue that needs attention is the problem of rare alleles and the corresponding rare variants, which need to be eliminated from the analysis involving GWAS due to statistical reasons. These rare variants may sometimes represent the most important variants, since desirable variants are expected to occur at a very low frequency. This is borne out by several studies including the recent study, where a rare allele of grain size gene *GS2* was identified to increase grain size and yield in rice [66]. During the present study also, we came across 316 rare alleles belonging to 165 SSRs. An examination of the rare variants for individual traits carrying these rare alleles suggests that at least some of these rare variants might carry desirable rare alleles for important QTL. Such possible candidates could be identified for 10 of the 14 traits. The desirable rare alleles and the corresponding rare variants for these 10 traits are listed in S3 Table. The significance of these rare variants can be exemplified by using the trait 1000-grain weight (TGW), for which some of the rare alleles (e.g., wmc652-148, cfa-2262-182, wmc405-121) appear to be important, since the rare variants carrying these rare alleles had a TGW ranging from 38.29 to 48.5 g (for details, see S3 Table). Therefore, it is possible that due to exclusion of these rare alleles, some important MTAs might have escaped detection during analysis.

Despite the above, we feel that the importance of rare alleles and rare variants has perhaps been overemphasized in recent literature. Although rare alleles for all markers taken together may explain sizable proportion of genetic variation, but majority of rare alleles may not belong to a QTL for the trait of interest. Also, in order to study the rare marker alleles, an appropriate experimental set up is necessary, which either increases relative frequency of rare alleles or modify the statistical model that can deal with rare alleles. Some of the solutions, which may be used in future research, include the following: (i) use of biparental mapping population (derived from genotypes with rare alleles); (ii) combined linkage-association mapping; (iii) use of large population; (iv) conducting separate analysis for common variants (CWAS) and rare variants (RVAS) [67], (v) advanced statistical tests like burden test, variance component test, combined omnibus test [68] (for details, see Gupta et al. [69]).

## Conclusions

In majority of crops including wheat, the quantitative traits with continuous variation are often complex in nature and are controlled each by a large number of main effect and interacting loci. In the present study, we identified a number of MTAs involving each of the 14 different traits using SLST, MLMM and MTMM. Some of the associations simply confirmed the QTLs reported earlier. The role of epistatic interactions in the genetic control of all the traits was also deciphered. Desirable alleles and allele combinations (at the interacting loci) along with eight superior wheat genotypes were identified. The problem of rare alleles and rare variants has also been discussed utilizing the data on rare variants from the present study. We also conclude from the present study that perhaps a combination of linkage analysis and association mapping could be the best approach for detecting maximum number of MTAS that are more robust and can be profitably utilized in molecular breeding.

## Supporting Information

S1 Table Correlation coefficient values for all possible pairs involving 14 traits. * and** indicate significance at 0.05 and 0.01 levels, respectively. Trait-pair showing correlation coefficient value ≥ 0.25 were used in multi-trait analysis and are highlighted in bold.
(DOCX)

S2 Table Mean squared differences (MSD) between observed and expected p-values for 14 traits using different models of association mapping. (DOCX)

S3 Table List of putative important rare alleles for 10 traits, along with range and mean trait value in rare variant and number of genotypes with respective rare allele. (DOCX)

S4 Table 56 important MTAs identified by different GWAS approaches along with interacting loci. (DOCX)

## References

[1] Remington DL, Thornsberry J, Matsuoka Y, Wilson L, Rinehart-Whitt S, Doebley J, et al Structure of linkage disequilibrium and phenotypic associations in the maize genome. Proc Natl Acad Sci USA. 2001; 98:11479–11484. 11562485 10.1073/pnas.201394398 PMC58755

[2] Falush D, Stephens M, Pritchard JK. Inference of population structure using multilocus genotype data: linked loci and correlated allele frequencies. Genetics. 2003; 164:1567–1587. 12930761 10.1093/genetics/164.4.1567 PMC1462648

[3] Buckler ES, Holland JB, Bradbury PJ, Acharya CB, Brown PJ, Browne C, et al The genetic architecture of maize flowering time. Science. 2009; 325:714–718. 10.1126/science.1174276 19661422

[4] Huang X, Wei X, Sang T, Zhao Q, Feng Q, Zhao Y, et al Genome-wide association studies of 14 agronomic traits in rice landraces. Nat Genet. 2010;42:961–969. 10.1038/ng.695 20972439

[5] Cai D, Xiao Y, Yang W, Ye W, Wang B, Younas M, et al. Association mapping of six yield-related traits in rapeseed (Brassica napus L.). Theor Appl Genet. 2014; 127:85–96. 24121524 10.1007/s00122-013-2203-9

[6] Tadesse W, Ogbonnaya FC, Jighly A, Sanchez-Garcia M, Sohail Q, Rajaram S. Genome-wide association mapping of yield and grain quality traits in winter wheat genotypes. PLoS One. 2015; 10: e0141339 10.1371/journal.pone.0141339 26496075 PMC4619745

[7] Atwell S, Huang YS, Vilhjálmsson BJ, Willems G, Horton M, Li Y, et al Genome-wide association study of 107 phenotypes in a common set of *Arabidopsis thaliana* inbred lines. Nature. 2010; 465:627–631. 10.1038/nature08800 20336072 PMC3023908

[8] Neumann K, Kobiljski B, Dencic S, Varshney RK, Borner A. Genome-wide association mapping: a case study in bread wheat (*Triticum aestivum* L.). Mol Breed. 2010; 27:37–58.

[9] Reif JC, Gowda M, Maurer HP, Longin CFH, Korzun V, Ebmeyer E, et al Association mapping for quality traits in soft winter wheat. Theor Appl Genet. 2011a; 122: 961–970. 21153626 10.1007/s00122-010-1502-7

[10] Reif JC, Maurer HP, Korzun V, Ebmeyer E, Miedaner T, Wurschum T. Mapping QTLs with main and epistatic effects underlying grain yield and heading time in soft winter wheat. Theor Appl Genet. 2011b; 123: 283–292. 21476040 10.1007/s00122-011-1583-y

[11] Mir RR, Kumar N, Jaiswal V, Girdharwal N, Prasad M, Balyan HS, et al Genetic dissection of grain weight in bread wheat through quantitative trait locus interval and association mapping. Mol Breed. 2012; 29: 963–972.

[12] Lopes MS, Dreisigacker S, Pena RJ, Sukumaran S, Reynolds MP. Genetic characterization of the wheat association mapping initiative (WAMI) panel for dissection of complex traits in spring wheat. Theor Appl Genet. 2015; 128: 453–464. 10.1007/s00122-014-2444-2 25540818

[13] Breseghello F, Sorrells ME. Association mapping of kernel size and milling quality in wheat (*Triticum aestivum* L.) cultivars. Genetics. 2006; 172:1165–1177. 16079235 10.1534/genetics.105.044586 PMC1456215

[14] Ravel C, Praud S, Murigneux A, Linossier L, Dardevet M, Balfourier F, et al Identification of *Glu-B1-1* as a candidate gene for the quantity of high-molecular-weight glutenin in bread wheat (*Triticum aestivum* L.) by means of an association study. Theor Appl Genet. 2006; 112:738–743. 16362275 10.1007/s00122-005-0178-x

[15] Tommasini L, Schnurbusch T, Fossati D, Mascher F, Keller B. Association mapping of *Stagonospora nodorum* blotch resistance in modern European winter wheat varieties. Theor Appl Genet. 2007; 115:697–708. 17634916 10.1007/s00122-007-0601-6

[16] Crossa J, Burgueño J, Dreisigacker S, Vargas M, Herrera-Foessel SA, Lillemo M, et al Association analysis of historical bread wheat germplasm using additive genetic covariance of relatives and population structure. Genetics. 2007; 177:1889–1913. 17947425 10.1534/genetics.107.078659 PMC2147943

[17] Gurung S, Mamidi S, Bonman JM, Xiong M, Brown-Guedira G, Adhikari TB. Genome-wide association study reveals novel quantitative trait loci associated with resistance to multiple leaf spot diseases of spring wheat. PLoS One. 2014; 9: e108179 10.1371/journal.pone.0108179 25268502 PMC4182470

[18] Gouis JL, Bordes J, Ravel C, Heumez E, Faure S, Praud S, et al Genome-wide association analysis to identify chromosomal regions determining components of earliness in wheat. Theor Appl Genet. 2011; 124:597–611. 10.1007/s00122-011-1732-3 22065067

[19] Maccaferri M, Sanguineti MC, Demontis A, Ahmed AE, Moral LG, Maalouf F, et al Association mapping in durum wheat grown across a broad range of water regimes. J Exp Bot. 2011; 62:409–438. 10.1093/jxb/erq287 21041372

[20] Maccaferri M, Zhang J, Bulli P, Abate Z, Chao S, Cantu D, et al A genome-wide association study of resistance to stripe rust (*Puccinia striiformis* f. sp. *tritici*) in a worldwide collection of hexaploid spring wheat (*Triticum aestivum* L.). G3. 2015; 20:449–465. 10.1534/g3.114.014563 PMC4349098 25609748

[21] Sukumaran S, Dreisigacker S, Lopes M, Chavez P, Reynolds MP. Genome-wide association study for grain yield and related traits in an elite spring wheat population grown in temperate irrigated environments. Theor Appl Genet. 2015; 128:353–363. 10.1007/s00122-014-2435-3 25490985

[22] Jaiswal V, Mir RR, Mohan A, Balyan HS, Gupta PK. Association mapping for pre-harvest sprouting tolerance in common wheat (*Triticum aestivum* L.). Euphytica. 2012; 188:89–102.

[23] Kulwal PL, Ishikawa G, Benscher D, Feng Z, Yu LX, Jadhav A, et al Association mapping for pre-harvest sprouting resistance in white winter wheat. Theor appl Genet. 2912; 125:793–805. 10.1007/s00122-012-1872-0 22547141

[24] Rehman AMA, Neumann K, Nagel M, Kobiljski B, Lohwasser U, Börner A. An association mapping analysis of dormancy and pre-harvest sprouting in wheat. Euphytica. 2012; 188:409–417.

[25] Liu Y, Wang L, Mao S, Liu K, Lu Y, Wang J, et al Genome-wide association study of 29 morphological traits in Aegilops tauschii. Sci Rep. 2016; 5:155–162. 10.1038/srep15562 PMC4622089 26503608

[26] Kulwal PL, Kumar N, Kumar A, Gupta RK, Balyan HS, Gupta PK. Gene networks in hexaploid wheat: interacting quantitative trait loci for grain protein content. Funct Integr Genomics. 2015; 5:254–259. 10.1007/s10142-005-0136-3 15711959

[27] Mohan A, Kulwal PL, Singh R, Kumar V, Mir RR, Kumar J, et al Genome-wide QTL analysis for pre-harvest sprouting tolerance in bread wheat. Euphytica. 2009; 168:319–329.

[28] Segura V, Vilhjalmsson BJ, Platt A, Korte A, Seren U, Long Q, et al An efficient multi-locus mixed-model approach for genomewide association studies in structured populations. Nat Genet. 2012; 44: 825–830. 10.1038/ng.2314 22706313 PMC3386481

[29] Korte A, Vilhjálmsson BJ, Segura V, Platt A, Long Q, Nordborg M. A mixed-model approach for genome-wide association studies of correlated traits in structured populations. Nat Genet. 2012; 44: 1066–1071. 10.1038/ng.2376 22902788 PMC3432668

[30] Mir RR, Kumar J, Balyan HS, Gupta PK. A study of genetic diversity among Indian bread wheat (Triticum aestivum L.) cultivars released during last 100 years. Genet Resour Crop Ev. 2012; 59: 717–726.

[31] Kundu S, Shoran J, Mishra B, Gupta RK. Indian wheat varieties at a glance. Directorate of Wheat Research, Karnal-132001, India. Research Bulletin No. 21; 2006.

[32] Pritchard JK, Stephens M, Donnelly P. Inference of population structure using multilocus genotype data. Genetics. 2000; 155:945–959. 10835412 10.1093/genetics/155.2.945 PMC1461096

[33] Stich B, Mohring J, Piepho HP, Heckenberger M, Buckler ES, Melchinger AE. Comparison of mixed-model approaches for association mapping. Genetics. 2008; 178: 1745–1754. 10.1534/genetics.107.079707 18245847 PMC2278052

[34] Kang HM, Ye C, Eskin E. Accurate discovery of expression quantitative trait loci under confounding from spurious and genuine regulatory hotspots. Genetics. 2008; 180:1909–1925. 10.1534/genetics.108.094201 18791227 PMC2600931

[35] Benjamini Y, Hochberg Y. Controlling the false discovery rate: a practical and powerful approach to multiple testing. J R Stat Soc. 1995; 57:289–300.

[36] Benson J, Brown-Guedira G, Murphy JP, Sneller C. Population structure, linkage disequilibrium, and genetic diversity in soft winter wheat enriched for fusarium head blight resistance. Plant Genome. 2012; 5:71–80.

[37] Gonzalez JR, Armengol L, Sole X, Guino E, Mercader JM, Estivill X, et al SNPassoc: an R package to perform whole genome association studies. Bioinformatics. 2007; 23:654–655. 17267436 10.1093/bioinformatics/btm025

[38] Thornsberry JM, Goodman MM, Doebley J, Kresovich S, Nielsen D, Buckler ES. *Dwarf8* polymorphisms associate with variation in flowering time. Nat Genet. 2001; 28:286–289. 11431702 10.1038/90135

[39] Larsson SJ, Lipka AE, Buckler ES. Lessons from Dwarf8 on the strengths and weaknesses of structured association mapping. PLoS Genet. 2013; 9:e1003246 10.1371/journal.pgen.1003246 23437002 PMC3578782

[40] Flint-Garcia SA, Thuillet AC, Yu J, Pressoir G, Romero SM, Sharon EM, et al Maize association population: a high-resolution platform for quantitative trait locus dissection. Plant J. 2005; 44:1054–1064. 16359397 10.1111/j.1365-313X.2005.02591.x

[41] Maccaferri M, Sanguineti MC, Mantovani P, Demontis A, Massi A, Ammar K, et al Association mapping of leaf rust response in durum wheat. Mol Breed. 2010; 26:189–228.

[42] Dodig D, Zoric M, Kobiljski B, Savic J, Kandic V, Quarrie S, et al Genetic and association mapping study of wheat agronomic traits under contrasting water regimes. Int J Mol Sci. 2012; 13:6167–6188. 10.3390/ijms13056167 22754357 PMC3382799

[43] Qian HR, Huang S. Comparison of false discovery rate methods in identifying genes with differential expression. Genomics. 2005; 86:495–503. 16054333 10.1016/j.ygeno.2005.06.007

[44] Huang XQ, Coster H, Ganal MW, Roeder MS. Advanced backcross QTL analysis for the identification of quantitative trait loci alleles from wild relatives of wheat (*Triticum aestivum* L.). Theor Appl Genet. 2003; 106:1379–1389. 12750781 10.1007/s00122-002-1179-7

[45] Huang XQ, Cloutier S, Lycar L, Radovanovic N, Humphreys DG, Noll JS, et al Molecular detection of QTLs for agronomic and quality traits in a doubled haploid population derived from two Canadian wheats (*Triticum aestivum* L.). Theor Appl Genet. 2006; 113:753–766. 16838135 10.1007/s00122-006-0346-7

[46] Quarrie SA, Steed A, Calestani C, Semikhodskii A, Lebreton C, Chinoy C, et al A high-density genetic map of hexaploid wheat (Triticum aestivum L.) from the cross Chinese Spring × SQ1 and its use to compare QTLs for grain yield across a range of environments. Theor Appl Genet. 2005; 110:865–880. 15719212 10.1007/s00122-004-1902-7

[47] Yao J, Wang L, Liu L, Zhao C, Zheng Y. Association mapping of agronomic traits on chromosome 2A of wheat. Genetica. 2009; 137:67–75. 10.1007/s10709-009-9351-5 19160058

[48] Zhang LY, Liu DC, Guo XL, Yang WL, Sun JZ, Wang DW, et al Genomic distribution of quantitative trait loci for yield and yield-related traits in common wheat. J Int Plant Biol. 2010a; 52:996–1007. 10.1111/j.1744-7909.2010.00967.x 20977657

[49] Zhang ZW, Ersoz E, Lai CQ, Fodhunter RJ, Tiwari HK, Gore MA, et al Mixed linear model approach adapted for genome-wide association studies. Nat Genet. 2010b; 42:355–360. 20208535 10.1038/ng.546 PMC2931336

[50] Zhang D, Hao C, Wang L, Zhang X. Identifying loci influencing grain number by microsatellite screening in bread wheat (Triticum aestivum L.). Planta. 2012; 236:1507–1517. 10.1007/s00425-012-1708-9 22820969

[51] Wang L, Ge H, Hao C, Dong Y, Zhang X. Identifying loci influencing 1,000-kernel weight in wheat by microsatellite screening for evidence of selection during Breeding. PLoS One. 2012; 7(2):e29432 10.1371/journal.pone.0029432 22328917 PMC3273457

[52] Edae EA, Byrne PF, Haley SD, Lopes MS, Reynolds MP. Genome-wide association mapping of yield and yield components of spring wheat under contrasting moisture regimes. Theor Appl Genet. 2014; 27:791–807. 10.1007/s00122-013-2257-8 24408378

[53] Gao H, Zhang T, Wu Y, Wu Y, Jiang L, Zhan J, et al Multiple-trait genome-wide association study based on principal component analysis for residual covariance matrix. Heredity. 2014; 113: 526–532. 10.1038/hdy.2014.57 24984606 PMC4274615

[54] Furlotte NA, Eskin E. Efficient multiple-trait association and estimation of genetic correlation using the matrix-variate linear mixed model. Genetics. 2015; 200:59–68. 10.1534/genetics.114.171447 25724382 PMC4423381

[55] Boone C, Bussey H, Andrews BJ. Exploring genetic interactions and networks with yeast. Nat Rev Genet. 2017; 8:437–449. 10.1038/nrg2085 17510664

[56] Phillips PC. Epistasis–the essential role of gene interactions in the structure and evolution of genetic systems. Nat Rev Genet. 2008; 9:855–867. 10.1038/nrg2452 18852697 PMC2689140

[57] Wang D, Eskridge KM, Crossa J. Identifying QTLs and epistasis in structured plant populations using adaptive mixed LASSO. J Agri Env Stat. 2010; 16:170–184.

[58] Kao CH, Zeng ZB, Teasdale RD. Multiple interval mapping for quantitative trait loci. Genetics. 1999; 152: 1203–1216. 10388834 10.1093/genetics/152.3.1203 PMC1460657

[59] Cantor RM, Lange K, Sinsheimer JS. Prioritizing GWAS results: a review of statistical methods and recommendations for their application. Am J Hum Genet. 2010; 86:6–22. 10.1016/j.ajhg.2009.11.017 20074509 PMC2801749

[60] Yu LX, Lorenz A, Rutkoski J, Singh RP, Bhavani S, Huerta-Espino J, et al Association mapping and gene–gene interaction for stem rust resistance in CIMMYT spring wheat germplasm. Theor Appl Genet. 2011; 123:1257–1268. 10.1007/s00122-011-1664-y 21811818

[61] Yu LX, Morgounov A, Wanyera R, Keser M, Singh SK, Sorrells M. Identification of *Ug99* stem rust resistance loci in winter wheat germplasm using genome-wide association analysis. Theor Appl Genet. 2012; 125:749–758. 10.1007/s00122-012-1867-x 22534791

[62] Langer SM, Friedrich C, Longin H, Wurschum T. Flowering time control in European winter wheat. Front Plant Sci. 2014; 5: 537 10.3389/fpls.2014.00537 25346745 PMC4191279

[63] Yang J, Hu C, Hu H, Yu R, Xia Z, Ye X, et al QTLNetwork: mapping and visualizing genetic architecture of complex traits in experimental populations. Bioinformatics. 2008; 24:721–723. 10.1093/bioinformatics/btm494 18202029

[64] Wang J, Joshi T, Valliyodan B, Shi H, Liang Y, Nguyen HT, et al A Bayesian model for detection of high order interactions among genetic variants in genome-wide association studies. BMC Genomics. 2015; 16:1011 10.1186/s12864-015-2217-6 26607428 PMC4660815

[65] Ruengphayak S, Chaichumpoo E, Phromphan S, Kamolsukyunyong W, Sukhaket W, Phuvanartnarubal E, et al Pseudo-backcrossing design for rapidly pyramiding multiple traits into a preferential rice variety. Rice. 2015; 8:7 10.1186/s12284-014-0035-0 25844112 PMC4384721

[66] Hu J, Wang Y, Fang Y, Zeng L, Xu J, Yu H, et al A rare allele of GS2 enhances grain size and grain yield in rice. Mol Plant. 2015; 8:1455–1465 10.1016/j.molp.2015.07.002 26187814

[67] Zuka O, Schaffnera SF, Samochaa K, Doa R, Hechtera E, Kathiresana S, et al Searching for missing heritability: desining rare variant association studies. Proc Natl Acad Sci USA. 2014; 111: E455–E464. 10.1073/pnas.1322563111 24443550 PMC3910587

[68] Lee S, Abecasis GR, Boehnke M, Lin X. Rare-variant association analysis: study designs and statistical tests. Am J Hum Genet. 2014; 95:5–23. 10.1016/j.ajhg.2014.06.009 24995866 PMC4085641

[69] Gupta PK, Kulwal PL, Jaiswal V. Association mapping in crop plants: opportunities and challenges. Adv Genet. 2014; 85: 109–148. 10.1016/B978-0-12-800271-1.00002-0 24880734

[70] Wang RX, Hai L, Zhang XY, You GX, Yan CS, Xiao SH. QTL mapping for grain filling rate and yield-related traits in RILs of the Chinese winter wheat population Heshangmai × Yu8679. Theor Appl Genet. 2009; 118:313–325. 10.1007/s00122-008-0901-5 18853131

[71] Patil RM, Tamhankar SA, Oak MD, Raut AL, Honrao BK, Rao VS, et al Mapping of QTL for agronomic traits and kernel characters in durum wheat (Triticum durum Desf.). Euphytica. 2013; 190:117–129.

[72] Peng J, Ronin Y, Fahima T, Roder MS, Li Y, Nevo E, et al Domestication quantitative trait loci in *Triticum dicoccoides*, the progenitor of wheat. Proc Natl Acad Sci USA. 2003; 100:2489–2494. 12604784 10.1073/pnas.252763199 PMC151368

[73] Yang DL, Jing RL, Chang XP, Li W. Identification of quantitative trait loci and environmental interactions for accumulation and remobilization of water-soluble carbohydrates in wheat (Triticum aestivum L.) stems. Genetics. 2007; 176:571–584. 17287530 10.1534/genetics.106.068361 PMC1893045

[74] Gupta PK, Rustig S, Kumar N. Genetic and molecular basis of grain size and grain number and its relevance to grain productivity in higher plants. Genome. 2006; 49:565–571. 16936836 10.1139/g06-063

[75] Sun XC, Marza F, Ma HX, Carver BF, Bai GH. Mapping quantitative trait loci for quality factors in an inter-class cross of US and Chinese wheat. Theor Appl Genet. 2010; 120:1041–1051. 10.1007/s00122-009-1232-x 20012855

[76] Somers DJ, Isaac P, Edwards K. A high-density microsatellite consensus map for bread wheat (Triticum aestivum L.). Theor Appl Genet. 2004; 109:1105–1114. 15490101 10.1007/s00122-004-1740-7

