# Supplementary material for: Genome Wide Single Locus Single Trait, Multi-Locus and Multi-Trait Association Mapping for Some Important Agronomic Traits in Common Wheat (T. aestivum L.)
Source: PLoS One. 2016 Jul 21;11(7):e0159343. doi: 10.1371/journal.pone.0159343 (PMC4956103; doi:10.1371/journal.pone.0159343)
Supplement: S1 Table — * and** indicate significance at 0.05 and 0.01 levels, respectively. Trait-pair showing correlation coefficient value ≥ 0.25 were used in multi-trait analysis and are highlighted in bold. (DOCX) [file pone.0159343.s001.docx]

**S1 Table.** Correlation coefficient values for all possible pairs involving 14 traits. * and** indicate significance at 0.05 and 0.01 level respectively. Trait-pair showing correlation coefficient value ≥ 0.25 are used in multi-trait analysis are highlighted in bold.

|  | PH | PL | FLL | AL | DTH | DTM | SL | SKS | GS | TGW | GPC | HI | HW | SV |
| --- | --- | --- | --- | --- | --- | --- | --- | --- | --- | --- | --- | --- | --- | --- |
| PH | 1 | **.661^**^** | .001 | -.131^*^ | **.276^**^** | **.399^**^** | -.060 | -.016 | **-.302^**^** | **.277^**^** | .180^**^ | -.220^**^ | .087 | **.266^**^** |
| PL |  | 1 | .132^*^ | .000 | -.105 | .012 | -.154^*^ | -.197^**^ | **-.406^**^** | .223^**^ | .059 | -.103 | .110 | .004 |
| FLL |  |  | 1 | .243^**^ | .060 | .025 | .**415^**^** | .146^*^ | .237^**^ | .000 | .003 | .041 | .047 | -.050 |
| AL |  |  |  | 1 | -.028 | .014 | -.006 | .004 | .136^*^ | .069 | -.107 | .136^*^ | .003 | -.130 |
| DTH |  |  |  |  | 1 | **.689^**^** | .214^**^ | **.524^**^** | **.298^**^** | -.072 | .020 | .091 | -.150^*^ | .025 |
| DTM |  |  |  |  |  | 1 | .197^**^ | **.355^**^** | .186^**^ | .159^*^ | -.046 | -.053 | .003 | .127 |
| SL |  |  |  |  |  |  | 1 | **.393^**^** | **.274^**^** | .068 | -.053 | -.002 | -.129 | -.066 |
| SKS |  |  |  |  |  |  |  | 1 | **.600^**^** | **-.315^**^** | -.083 | .207^**^ | -.147^*^ | -.080 |
| GS |  |  |  |  |  |  |  |  | 1 | **-.397^**^** | -.149^*^ | .235^**^ | -.041 | -.118 |
| TGW |  |  |  |  |  |  |  |  |  | 1 | .058 | **-.319^**^** | .170^*^ | .072 |
| GPC |  |  |  |  |  |  |  |  |  |  | 1 | -.048 | .040 | .154^*^ |
| HI |  |  |  |  |  |  |  |  |  |  |  | 1 | -.068 | **-.432^**^** |
| HW |  |  |  |  |  |  |  |  |  |  |  |  | 1 | -.213^**^ |
| SV |  |  |  |  |  |  |  |  |  |  |  |  |  | 1 |
