# Supplementary material for: Genome Wide Single Locus Single Trait, Multi-Locus and Multi-Trait Association Mapping for Some Important Agronomic Traits in Common Wheat (T. aestivum L.)
Source: PLoS One. 2016 Jul 21;11(7):e0159343. doi: 10.1371/journal.pone.0159343 (PMC4956103; doi:10.1371/journal.pone.0159343)
Supplement: S2 Table — (DOCX) [file pone.0159343.s002.docx]

**S2 Table.** Mean squared differences (MSD) between observed and expected p-values for 14 traits using different models of association mapping.

| Traits | Mean of the squared differences (MSD) | | | | Best fit model (minimum MSD) |
| --- | --- | --- | --- | --- | --- |
|  | Naive | Q | K | Q + K |  |
| PH | 0.14502 | 0.04009 | 0.00052 | 0.00076 | K |
| PL | 0.09210 | 0.02553 | 0.00098 | 0.00091 | Q + K |
| FLL | 0.03009 | 0.00734 | 0.00019 | 0.00034 | K |
| AL | 0.02073 | 0.01545 | 0.00313 | 0.00330 | K |
| DTH | 0.01926 | 0.00583 | 0.00023 | 0.00038 | K |
| DTM | 0.00867 | 0.00438 | 0.00074 | 0.00045 | Q + K |
| SL | 0.05131 | 0.00696 | 0.00018 | 0.00021 | K |
| SKS | 0.06162 | 0.00892 | 0.00037 | 0.00056 | K |
| GS | 0.11725 | 0.02100 | 0.00022 | 0.00027 | K |
| TGW | 0.04772 | 0.02184 | 0.00145 | 0.00117 | Q + K |
| GPC | 0.03457 | 0.00316 | 0.00184 | 0.00306 | K |
| HI | 0.02863 | 0.01003 | 0.00162 | 0.00110 | Q + K |
| HW | 0.00250 | 0.00236 | 0.00483 | 0.00410 | Q |
| SV | 0.03344 | 0.00037 | 0.00349 | 0.00029 | Q + K |
