# Supplementary material for: Genome Wide Single Locus Single Trait, Multi-Locus and Multi-Trait Association Mapping for Some Important Agronomic Traits in Common Wheat (T. aestivum L.)
Source: PLoS One. 2016 Jul 21;11(7):e0159343. doi: 10.1371/journal.pone.0159343 (PMC4956103; doi:10.1371/journal.pone.0159343)
Supplement: S3 Table — (DOCX) [file pone.0159343.s003.docx]

**S3 Table.** List of putative important rare alleles for 10 traits, along with range and mean trait value in rare variant and number of genotypes with respective rare allele.

| Trait (trait value in population) | No of Important rare alleles | Rare allele | No of genotypes with rare allele | Range trait value in rare variants | Mean trait value in rare variants |
| --- | --- | --- | --- | --- | --- |
| AL (0-11.59mm) | 7 | gwm413-96 | 3 | 7.63 - 11.31 | 9.56 |
|  |  | wmc94-127 | 7 | 8.25 - 11.59 | 9.13 |
|  |  | cfd23-null | 5 | 7.72 - 11.31 | 9.05 |
|  |  | wmc603-247 | 2 | 9.17 - 9.72 | 9.44 |
|  |  | gwm371-216 | 2 | 9.17 - 9.45 | 9.31 |
|  |  | barc24-165 | 3 | 8.94 - 9.75 | 9.38 |
|  |  | wmc233-260 | 4 | 7.01 - 10.16 | 9.16 |
| DTH (84-107) | 17 | wmc216-null | 2 | 85 - 93 | 89.00 |
|  |  | wmc474-119 | 6 | 88 - 95 | 89.83 |
|  |  | wmc527-365 | 2 | 85 - 88 | 86.50 |
|  |  | wmc652-163 | 9 | 84 - 93 | 88.89 |
|  |  | wmc94-127 | 7 | 85 - 95 | 89.71 |
|  |  | barc68-null | 11 | 87 - 98 | 91.36 |
|  |  | gwm558-214 | 11 | 86 - 98 | 90.36 |
|  |  | gwm294-100 | 6 | 84 - 97 | 88.50 |
|  |  | wmc285-354 | 3 | 86 - 88 | 87.00 |
|  |  | wmc48-130 | 4 | 85 - 90 | 88.50 |
|  |  | wmc405-null | 2 | 87 - 89 | 88.00 |
|  |  | wmc473-null | 4 | 87 - 90 | 88.75 |
|  |  | wmc372-192 | 2 | 89 - 90 | 89.50 |
|  |  | wmc603-243 | 7 | 85 - 96 | 89.42 |
|  |  | wmc498-null | 3 | 84 -92 | 87.00 |
|  |  | wmc307-198 | 2 | 86- 86 | 86.00 |
|  |  | barc24-164 | 10 | 85 - 98 | 89.70 |
| FLL (22.28-36.82) | 9 | wmc318-null | 2 | 32.41 - 32.8 | 32.60 |
|  |  | wmc335-110 | 4 | 28.3 - 33.34 | 31.04 |
|  |  | wmc494-220 | 3 | 30.55 - 33.40 | 31.96 |
|  |  | wmc419-170 | 5 | 29.08 - 32.81 | 31.54 |
|  |  | wmc285-354 | 3 | 30.65 - 33.17 | 31.87 |
|  |  | gwm135-260 | 2 | 31.21 - 31.2 | 31.20 |
|  |  | wmc372-234 | 7 | 29.1 - 36.4 | 31.57 |
|  |  | barc24-165 | 3 | 30.57 - 33.4 | 31.72 |
|  |  | barc24-172 | 3 | 30.02 - 33.26 | 31.23 |
| GPC (9.57-15.32%) | 9 | wmc245-157 | 5 | 12.2 - 14.01 | 12.99 |
|  |  | wmc311-null | 3 | 12.58 - 13.62 | 13.22 |
|  |  | wmc396-180 | 6 | 12.2 - 13.84 | 12.99 |
|  |  | wmc474-119 | 6 | 11.23 - 14.31 | 13.14 |
|  |  | wmc473-133 | 4 | 13.03 - 14.01 | 13.29 |
|  |  | gwm135-106 | 3 | 12.43 - 15.32 | 13.48 |
|  |  | gwm135-260 | 2 | 12.6 - 14.09 | 13.34 |
|  |  | wmc626-149 | 8 | 11.97 - 15.32 | 13.62 |
|  |  | gwm149-261 | 7 | 12.5 - 13.94 | 13.18 |
| HI (33-110.6) | 6 | gwm191-null | 2 | 85.7 - 93.3 | 89.50 |
|  |  | wmc422-303 | 2 | 82.1 - 90.8 | 86.45 |
|  |  | wmc93-99 | 2 | 81.9 - 89.2 | 85.55 |
|  |  | wmc89-239 | 2 | 82.1 - 89.1 | 85.60 |
|  |  | wmc372-192 | 2 | 83.2 - 90.8 | 87.00 |
|  |  | barc170-180 | 4 | 83.3 - 89.3 | 85.62 |
| HW (30-95.1) | 2 | wmc245-157 | 5 | 80.5 - 95.1 | 84.20 |
|  |  | wmc396-180 | 6 | 79.5 - 95.1 | 83.82 |
| PH (62-131) | 5 | wmc705-158 | 4 | 74 - 93 | 86.50 |
|  |  | gwm357-153 | 2 | 83 - 85 | 84.00 |
|  |  | wmc419-null | 2 | 80 - 81 | 80.50 |
|  |  | wmc285-354 | 3 | 83 - 89 | 85.67 |
|  |  | gwm293-158 | 2 | 62 - 90 | 76.00 |
| GS (36-81) | 7 | wmc500-null | 5 | 58 - 69 | 64.60 |
|  |  | wmc598-154 | 4 | 54 - 73 | 64.00 |
|  |  | barc5-null | 2 | 70 - 78 | 74.00 |
|  |  | wmc89-239 | 2 | 67 - 73 | 70.00 |
|  |  | wmc372-192 | 2 | 62 - 65 | 63.50 |
|  |  | gwm459-118 | 2 | 68 - 69 | 68.50 |
|  |  | wmc764-213 | 2 | 67 - 69 | 68.00 |
| SKS (14-26) | 3 | gwm191-null | 2 | 22 - 24 | 23.00 |
|  |  | gwm636-134 | 3 | 20 - 24 | 21.33 |
|  |  | gwm459-118 | 2 | 22 - 22 | 22.00 |
| TGW (31.1-48.5 g) | 13 | gwm131-147 | 3 | 38.49 -41.20 | 39.70 |
|  |  | gwm361-null | 2 | 39.44 - 42.12 | 40.78 |
|  |  | gwm469-null | 5 | 38.15 - 45.32 | 40.84 |
|  |  | wmc161-197 | 5 | 39.62 - 45.60 | 41.21 |
|  |  | wmc640-141 | 10 | 37.57 - 45.38 | 41.10 |
|  |  | wmc652-148 | 3 | 38.29 - 48.2 | 41.76 |
|  |  | cfa-2262-182 | 3 | 39.1 - 47.04 | 43.87 |
|  |  | wmc93-null | 2 | 42.85 - 45.00 | 43.92 |
|  |  | wmc109-189 | 2 | 41.64 - 42.64 | 42.14 |
|  |  | gwm296-142 | 5 | 38.01 - 45.88 | 41.63 |
|  |  | wmc405-121 | 4 | 41.2 - 48.5 | 44.44 |
|  |  | wmc283-175 | 11 | 38.02 - 48.5 | 41.53 |
|  |  | gwm539-150 | 6 | 38.02 - 47.04 | 41.19 |
