# Supplementary material for: Genome Wide Single Locus Single Trait, Multi-Locus and Multi-Trait Association Mapping for Some Important Agronomic Traits in Common Wheat (T. aestivum L.)
Source: PLoS One. 2016 Jul 21;11(7):e0159343. doi: 10.1371/journal.pone.0159343 (PMC4956103; doi:10.1371/journal.pone.0159343)
Supplement: S4 Table — (DOCX) [file pone.0159343.s004.docx]

**S4 Table.** 56 important MTAs identified by different GWAS approaches along with interacting loci.

| **GWAS Approach/Trait** | **Marker** | **Chro** | **Report of earlier studies** | **Interacting Loci** |
| --- | --- | --- | --- | --- |
| **SLST (qualified FDR)** | | | | |
| HI | gwm294 | 2A | [1-4] | psp3094 |
| HW | wmc598 | 2A | [5-6] | wmc474 |
| HW | wmc827 | 2A | [1] | - |
| HW | gwm459 | 6A | [1,7] | cfd62 |
| PH | gwm533.1 | 3B | [5] | - |
| SV | gwm111 | 7D | [8] |  |
| SV | wmc396 | 7B | [1] | wmc473a |
| SV, HI | gwm361 | 6B | [9] | wmc405 (for SV), gwm106 (for HI) |
| TGW | gwm107 | 3B | [1] |  |
| **SLST only (did not qualified FDR but reported in earlier studies)** | | | | |
| GS | wmc24 | 1A | [4, 10-11] | wmc626 gwm425 |
| GS | gwm413 | 1B |  |  |
| PH | gwm296 | 2A | [10] |  |
| PH | gwm349 | 2D | [10] |  |
| SL | wmc702 | 2A | [6] |  |
| TGW | gwm11 | 1B | [2, 8, 12-16] | cfd2 |
| TGW | barc164 | 3B |  | wmc475 |
| TGW | wmc593 | 7A |  |  |
| TGW | wmc516 | 4A |  |  |
| **MTMM only** | | | | |
| DTM | wmc764 | 2B | [17] | - |
| DTM | gwm276 | 7A | [18] | - |
| PH | gwm107 | 3B | [1] | - |
| PH | wmc764 | 2B | [17] | - |
| PH | wmc532 | 3A | [19] | - |
| PH | wmc396 | 7B | [1] | - |
| SV | gwm533.1 | 3B | [5] | - |
| SV | wmc233 | 5D | - | - |
| **SLST+MLMM** | | | | |
| HW | wmc827 | 2A | [1] | - |
| HW | wmc419 | 1B | [20] | wmc474, cfd62 |
| HW | wmc598 | 2A | [5-6] | wmc474 |
| HW | wmc313 | 4A | - | wmc474 , gwm636 |
| HW | gwm459 | 6A | - | cfd62 |
| AL | wmc597 | 1B | - | - |
| AL | wmc245 | 2B | - | - |
| AL | gwm480 | 3A | - | wmc396 |
| AL | gwm251 | 4B | - | cfd190a |
| AL | wmc75 | 5B | - | gwm302 |
| AL | wmc486 | 6B | - | - |
| GPC | wmc219 | 4A | [21] | wmc702, barc24 |
| **SLST+MTMM** | | | | |
| DTH | wmc764 | 2B | - | gwm99 , gwm44 |
| DTH | gwm276 | 7A | - | - |
| DTH | gwm44 | 7D | [22] | wmc764 |
| DTM | gwm294 | 2A | [1-4] | wmc473b |
| DTM | gwm66 | 4B | [23] | - |
| DTM | gwm44 | 7D | [22] | gwm149 |
| SL | psp3094 | 7D | - | - |
| SV | wmc245 | 2B | [24] | wmc405 |
| HI | wmc245 | 2B | [24] | - |
| HI | wmc532 | 3A | [19] | - |
| HI | wmc121 | 7D | - | - |
| PH | wmc598 | 2A | [5-6] | - |
| PH | wmc522 | 2A | - | - |
| PH | gwm294 | 2A | [1-4] | wmc219 |
| TGW | wmc48 | 4A | [25-27] | wmc364a |
| **MLMM+MTMM** | | | | |
| SV | gwm99 | 1A | [28] | - |
| SV | wmc764 | 2B | [17] | - |
| **SLST+MLMM+MTMM** | | | | |
| SV | wmc598 | 2A | [5-6] | wmc498 |
| SV | wmc532 | 3A | [19] | barc170 |
| SV | wmc396 | 7B | [1] | - |
| SV | wmc121 | 7D | - | wmc473 |
| HI | gwm294 | 2A | [1-4] | psp3094 |
| PH | gwm533.1 | 3B | [5] | - |
| TGW | gwm107 | 3B | [1] | - |

**References**

[1] Maccaferri M, Sanguineti MC, Demontis A, Ahmed AE, Moral LG, Maalouf F, et al. Association mapping in durum wheat grown across a broad range of water regimes. J Exp Bot. 2011; 62:409–438.

[2] Quarrie SA, Steed A, Calestani C, Semikhodskii A, Lebreton C, Chinoy C, et al. A high-density genetic map of hexaploid wheat (*Triticum aestivum* L.) from the cross Chinese Spring × SQ1 and its use to compare QTLs for grain yield across a range of environments. Theor Appl Genet. 2005; 110:865–880.

[3] Hu J, Wang Y, Fang Y, Zeng L, Xu J, Yu H, et al. A rare allele of *GS*2 enhances grain size and grain yield in rice. Mol Plant. 2015; doi:101016/jmolp201507002.

[4] Zhang D, Hao C, Wang L, Zhang X. Identifying loci influencing grain number by microsatellite screening in bread wheat (*Triticum aestivum* L.). Planta. 2012; 236:1507-1517.

[5] Wang RX, Hai L, Zhang XY, You GX, Yan CS, Xiao SH. QTL mapping for grain filling rate and yield-related traits in RILs of the Chinese winter wheat population Heshangmai × Yu8679. Theor Appl Genet, 2009; 118:313–325.

[6] Yao J, Wang L, Liu L, Zhao C, Zheng Y. Association mapping of agronomic traits on chromosome 2A of wheat. Genetica. 2009; 137:67–75.

[7] Yang DL, Jing RL, Chang XP, Li W. Identification of quantitative trait loci and environmental interactions for accumulation and remobilization of water-soluble carbohydrates in wheat (*Triticum aestivum* L.) stems. Genetics. 2007; 176:571–584.

[8] Mir RR, Kumar N, Jaiswal V, Girdharwal N, Prasad M, Balyan HS, et al. Genetic dissection of grain weight in bread wheat through quantitative trait locus interval and association mapping. Mol Breed. 2012; 29:963–972.

[9] Patil RM, Tamhankar SA, Oak MD, Raut AL, Honrao BK, Rao VS, et al. Mapping of QTL for agronomic traits and kernel characters in durum wheat (*Triticum durum* Desf.). Euphytica. 2013; 190:117-129.

[10] Zhang LY, Liu DC, Guo XL, Yang WL, Sun JZ, Wang DW, et al. Genomic distribution of quantitative trait loci for yield and yield-related traits in common wheat. J Int Plant Biol. 2010a; 52:996–1007.

[11] Zhang ZW, Ersoz E, Lai CQ, Fodhunter RJ, Tiwari HK, Gore MA, et al. Mixed linear model approach adapted for genome-wide association studies. Nat Genet. 2010b; 42:355–360.

[12] Huang XQ, Coster H, Ganal MW, Roeder MS. Advanced backcross QTL analysis for the identification of quantitative trait loci alleles from wild relatives of wheat (*Triticum* *aestivum* L.). Theor Appl Genet. 2003; 106:1379–1389.

[13] Huang XQ, Cloutier S, Lycar L, Radovanovic N, Humphreys DG, Noll JS, et al. Molecular detection of QTLs for agronomic and quality traits in a doubled haploid population derived from two Canadian wheats (*Triticum aestivum* L.). Theor Appl Genet. 2006; 113:753–766.

[14] Wang L, Ge H, Hao C, Dong Y, Zhang X. Identifying loci influencing 1,000-kernel weight in wheat by microsatellite screening for evidence of selection during Breeding. PLoS One. 2012; 7:e29432.

[15] Gupta PK, Rustig S, Kumar N. Genetic and molecular basis of grain size and grain number and its relevance to grain productivity in higher plants. Genome 2006; 49:565–571.

[16] Sun XC, Marza F, Ma HX, Carver BF, Bai GH. Mapping quantitative trait loci for quality factors in an inter-class cross of US and Chinese wheat. Theor Appl Genet. 2010; 120:1041–1051.

[17] Azadi A, Mardi M, Hervan EM, Mohammadi SA, Moradi F, Tabatabaee MT, et al. QTL mapping of yield and yield components under normal and salt-stress conditions in bread wheat (*Triticum aestivum* L.). Plant Mol Biol Rep. 2015; 33:102–120.

[18] Zhang W, Chao S, Manthey F, Chicaiza O, Brevis JC, Echenique V, et al. QTL analysis of pasta quality using a composite microsatellite and SNP map of durum wheat. Theor Appl Genet. 2008; 117:1361–1377.

[19] Zhang W, Zhang L, Qiao L, Wu J, Zhao G, Jing R, et al. Cloning and haplotypes analysis of *TaSTE*, which is associated with plant height in bread wheat (*Triticum aestivum* L.) Mol Breed. 2013; 31:47-56.

[20] Shahzada M, Khanb, Khana AS, Sajjadd M, Rehmanc A, Khana AI. Identification of QTLs on chromosome 1B for grain quality traits in bread wheat (*Triticum aestivum* L.). Cytol Genet. 2016; 50:89–95.

[21] Jordan MC, Somers DJ, Banks TW. Identifying regions of the wheat genome controlling seed development by mapping expression quantitative trait loci. Plant Biotechnol J. 2007; 5:442–453.

[22] Bonnin I, Rousset M, Madur D, Sourdille P, Dupuits C, Brunel D, et al. FT genome A and D polymorphisms are associated with the variation of earliness components in hexaploid wheat. Theor Appl Genet. 2008; 116:383–394.

[23] Mares D, Rathjen J, Mrva K, Cheong J. Genetic and environmental control of dormancy in white-grained wheat (*Triticum aestivum* L.). Euphytica. 2009; 168:311–318.

[24] Roy JK, Bandopadhyay R, Rustgi S, Balyan HS, Gupta PK. Association analysis of agronomically important traits using SSR, SAMPL and AFLP markers in bread wheat. Curr Sci. 2006; 90:683-689.

[25] Zhao J, Wang H, Zhang X, Du X, Li A, Kong L. Association analysis of grain traits with SSR markers between *Aegilops tauschii* and hexaploid wheat (*Triticum aestivum* L*.*) J Integ Agr 2015; doi10.1016/S2095-3119(15)61070-X.

[26] Barakat MN, Al-Doss AA, Elshafei1 AA, Moustafa KA. Bulked segregant analysis to detect quantitative trait loci (QTL) related to heat tolerance at grain filling rate in wheat using simple sequence repeat (SSR) markers. Afr J Biotechnol. 2012; 11:12436-12442.

[27] Brbaklic L, Trkulja D, Kondic-Spika A, Treskic S, Kobiljski B. Detection of QTLs for important agronomical traitsin hexaploid wheat using association analysis. Czech J Genet Plant Breed. 2013; 49:1–8.

[28] Dodig D, Zoric M, Kobiljski B, Savic J, Kandic V, Quarrie S, et al. Genetic and association mapping study of wheat agronomic traits under contrasting water regimes. Int J Mol Sci. 2012; 13:6167-6188.
